# An integrative neuropharmacological review of Huntington’s disease challenges and the role of novel formulations in addressing pharmacological‒pharmaceutical limitations

**DOI:** 10.3389/fphar.2026.1794983

**Published:** 2026-05-07

**Authors:** Mahmoud A. Senousy, Aya H. Eid, Mohamed Bakr Zaki, Mai A. Abd-Elmawla, Heba R. Ghaiad, Riham A. El-Shiekh, Sadek Ahmed, Hazim O. Khalifa

**Affiliations:** 1 Department of Biochemistry, Faculty of Pharmacy, Cairo University, Cairo, Egypt; 2 Department of Biochemistry, Faculty of Pharmacy and Drug Technology, Egyptian Chinese University, Cairo, Egypt; 3 Pharmacology and Toxicology Department, Faculty of Pharmacy, Heliopolis University, Cairo, Egypt; 4 Department of Biochemistry, Faculty of Pharmacy, University of Sadat City, Menoufia, Egypt; 5 Department of Biochemistry, Faculty of Pharmacy, Ahram Canadian University, 6th of October City, Egypt; 6 Department of Pharmacognosy, Faculty of Pharmacy, Cairo University, Cairo, Egypt; 7 Department of Pharmaceutics and Industrial Pharmacy, Faculty of Pharmacy, Cairo University, Cairo, Egypt; 8 Department of Veterinary Medicine, College of Agriculture and Veterinary Medicine, United Arab Emirates University, Al Ain, United Arab Emirates; 9 United Arab Emirates University (UAEU) Center for Public Policy and Leadership, United Arab Emirates University, Al Ain, United Arab Emirates

**Keywords:** disease modifiers, gut–brain axis, Huntington’s disease, immune dysregulation, molecular and cellular mechanisms, multisystem pathology, neurodegeneration, therapeutic implications

## Abstract

**Background:**

Huntington’s disease (HD) is an autosomal dominant neurodegenerative disorder caused by CAG repeat expansion in the huntingtin gene, leading to progressive neuronal dysfunction and neurodegeneration. Although classically defined as a brain-restricted disorder marked by striatal and cortical degeneration, increasing evidence suggests HD as a multisystem disease involving both central and peripheral pathological alterations.

**Objective:**

This review aims to provide an integrated overview of neuronal and non-neuronal mechanisms underlying HD, focusing on systemic alterations that influence disease onset, progression, and clinical variability. This review also aims to connect neuropharmacology with pharmaceutical formulation strategies, particularly emphasizing the therapeutic and drug-delivery challenges and nanotechnology-based solutions.

**Methods:**

A structured literature review was conducted using databases including PubMed, EMBASE, and Scopus. Using the appropriate keywords, original articles, clinical studies, systematic reviews, meta-analyses, and high-quality reviews were selected based on their relevance to HD pathophysiology and therapeutic strategies.

**Results:**

HD manifests with motor, cognitive, and psychiatric disturbances; however, this review highlights that peripheral immune activation, gut microbiota dysbiosis, and multiorgan pathology are not merely secondary features but interact with neural circuits, contributing to disease heterogeneity and progression. Current therapeutic approaches are largely symptomatic, achieving minimal effectiveness in disease modification due to challenges such as poor blood–brain barrier penetration, limited target selectivity, and inter-individual variability. New strategies, such as nanotechnology-based drug delivery systems, biologics, and gene editing tools, offer advantages and support a deeper understanding of therapeutic limitations and disease mechanisms, yet their translational applicability remains constrained by limited clinical validation, safety concerns, and scalability problems.

**Conclusion:**

Reconceptualizing HD as a multisystem disorder provides a more comprehensive framework for therapeutic development. Integrating central and peripheral disease mechanisms with advances in targeted drug delivery and patient stratification approaches, such as sex differences, hormonal influences, and environmental factors, is essential for translational progress toward personalized therapeutic approaches. Future research should prioritize interdisciplinary approaches to bridge the gap between mechanistic discoveries and effective disease-modifying interventions.

## 1 Introduction

Huntington’s disease (HD) is an autosomal dominant inherited neurodegenerative disorder that progressively worsens over time and is characterized by uncontrolled choreiform motions, such as jerking or writhing without conscious thought; dementia or cognitive deterioration, and psychiatric disorders (personality changes, irritability, and sadness) (Bates et al., 2014; Bates et al., 2015). HD is genetically caused by a mutation in the coding region of the huntingtin (HTT) gene, located at 4p16.3. This mutation results in CAG trinucleotide repeats (40 or more) and the subsequent development of a mutant HTT (mHTT) protein with an abnormally long polyglutamine (polyQ) tract. Accumulation of mHTT in the brain leads to advanced neuronal degeneration, especially in the basal ganglia (putamen and caudate nucleus) (Li et al., 2003; Burgunder, 2014).

There are two distinct clinical manifestations of HD: the classical choreic (hyperkinetic) form and the rigid (hypokinetic) form; each predominates at different stages of the disease. Clinically, the choreic type is the predominant and hallmark motor manifestation, particularly in the early to mid-stages, and is often characterized by abnormal involuntary movements (chorea), a range of mental disorders, and intellectual impairment (dementia). The overactivation of motor pathways and chorea results from the loss of inhibitory GABAergic neurons in the striatum and basal ganglia, whereas psychiatric symptoms and dementia are caused by progressive cortical involvement. As HD progresses, chorea is replaced by a hypokinetic-rigid form of HD (Bates et al., 2014; Bates et al., 2015).

Although previous reviews have extensively focused on the molecular mechanisms and therapeutic targets of HD, comparatively limited attention has been given to the critical interplay among disease biology, drug delivery challenges, and formulation-driven solutions. In particular, the integration of pathophysiological insights with advanced drug delivery strategies, especially nanotechnology-based systems, remains insufficiently explored, representing a key barrier to successful clinical translation.

In this context, the present review aims to provide a comprehensive and integrative perspective on HD by linking its molecular and systemic pathophysiology with emerging therapeutic strategies and formulation challenges. The review highlights not only classical neuronal degeneration but also non-neuronal mechanisms, including immune dysregulation, gut–brain axis alterations, and peripheral organ involvement, thereby underscoring the systemic nature of the disease. Furthermore, it discusses advances in experimental models, diagnostic challenges, and repurposed therapeutic agents while emphasizing the growing importance of innovative drug delivery approaches such as nanocarriers, biological therapeutics, and non-invasive administration routes.

**FIGURE 1:**
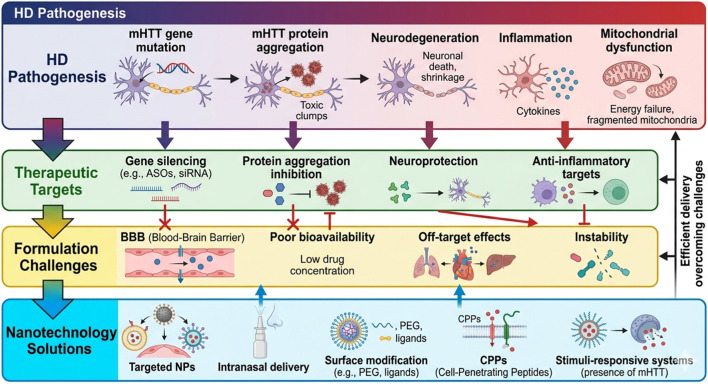
Integrated framework of Huntington’s disease pathogenesis, therapeutic targets, and nanotechnology-enabled solutions. The top panels depict the HD pathological cascade (purple) and associated therapeutic targets (green). The lower panels illustrate the biological barriers (yellow) that limit conventional drug delivery, alongside nanotechnology-enabled solutions (blue), including targeted nanoparticles and intranasal delivery, designed to overcome these barriers. Vertical arrows indicate the integrated flow from identifying disease mechanisms to developing efficient, site-specific therapeutic delivery systems. ASOs, antisense oligonucleotides; BBB, blood–brain barrier; CPPs, cell-penetrating peptides; HD, Huntington’s disease; mHTT, mutant Huntingtin; NPs, nanoparticles; PEG, polyethylene glycol; siRNA, small interfering RNA. This figure was generated with the assistance of AI on Google Search, which is powered by the Gemini family of models, based on content curated, reviewed, and validated by the authors.

Importantly, this review proposes that bridging HD pathogenesis with advanced formulation strategies, particularly nanotechnology-based delivery systems, represents a critical and underexplored approach to overcoming current translational barriers. An integrated framework linking HD pathogenesis, therapeutic targets, formulation challenges, and nanotechnology-enabled solutions is illustrated in Figure 1. By integrating molecular, systemic, and technological perspectives, this work provides a structured framework to guide future research and accelerate the development of effective and personalized therapeutic interventions for HD.

## 2 Methodology

A structured literature review was conducted using databases including PubMed, EMBASE, and Scopus, covering studies published up to 2026. Search terms combined “Huntington’s disease” with keywords such as “neuroinflammation,” “gut–brain axis,” “peripheral pathology,” “drug delivery,” and “nanotechnology.” Original research articles, clinical studies, systematic reviews, meta-analyses, and high-quality reviews were selected based on relevance to HD pathophysiology and therapeutic strategies, with priority given to recent and mechanistically informative studies.

## 3 Experimental HD models overview

**FIGURE 2:**
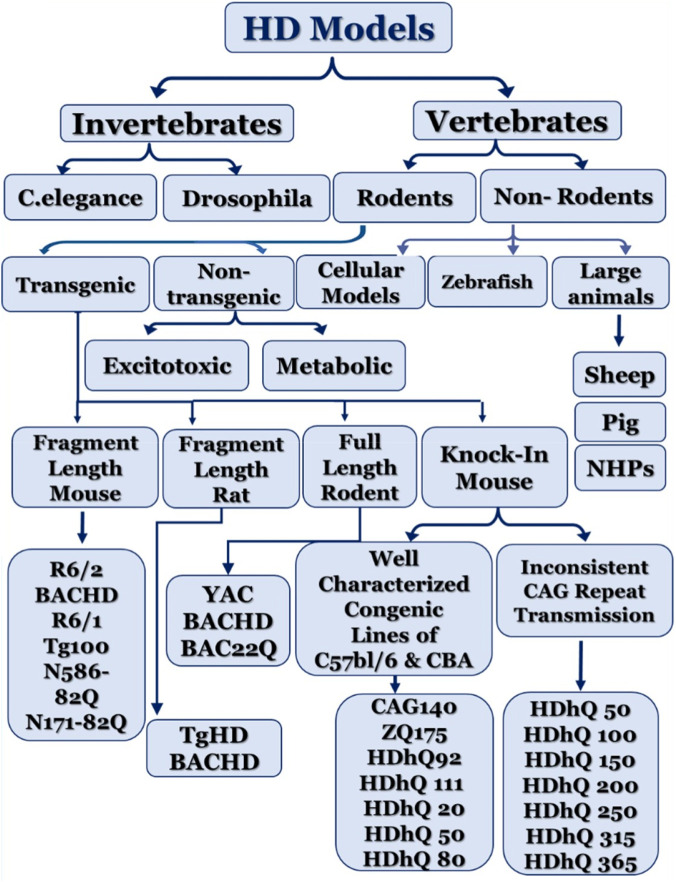
Schematic classification of the animal models developed for modeling HD (Salman et al., 2025).

**FIGURE 3:**
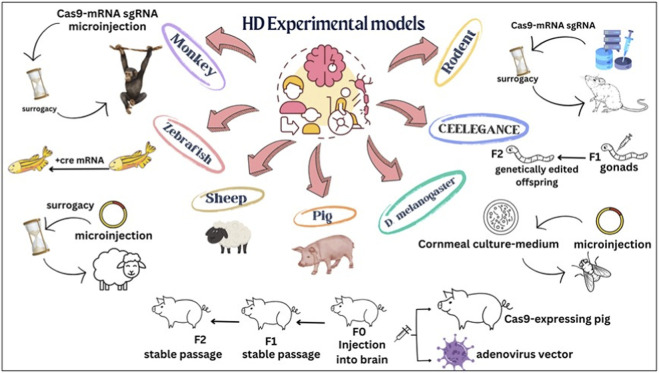
Various experimental models and modeling methods used in HD.

An ideal HD preclinical model should replicate key pathological, molecular, and behavioral features associated with mHTT, including progressive neurodegeneration (Nittari et al., 2023). Existing experimental models are broadly classified into invertebrate and vertebrate systems, each offering distinct advantages for mechanistic studies and drug discovery. A schematic classification of the experimental models developed for modeling HD is presented in Figure 2. Invertebrate models such as *C. elegans* and *Drosophila* enable high-throughput screening and investigation of polyglutamine toxicity; however, their limited neuroanatomical complexity restricts their translational applicability (Jackson et al., 1998; Brignull et al., 2006; Yu et al., 2024). This section focuses on the vertebrate, cellular, and alternative models of HD (Figure 3), their translational relevance, and limitations.

### 3.1 Non-genetic (toxin-induced) rodent models

Toxin-based models are widely used to mimic specific pathological mechanisms, particularly excitotoxicity and mitochondrial dysfunction. Common agents include quinolinic acid (QA), kainic acid, and 3-nitropropionic acid (3-NP), which induce selective striatal degeneration via N-methyl-D-aspartate (NMDA) receptor overactivation or mitochondrial impairment (Ramaswamy et al., 2007; Noureldeen et al., 2024). A comparative summary of toxin models of HD is provided in Table 1.

**TABLE 1:** Toxin-induced HD models.

Animal model	Species	Mode of administration	Method of cell death	Motor symptoms	Cognitive symptoms
QA	Rat and non-human primate	Intrastriatal injections, intraputamenal injections	Excitotoxicity	Hyperkinesia, apomorphine-induced dystonia and dyskinesia, spontaneous dyskinesia with higher doses	Visuospatial deficits, procedural memory deficits, poor memory recall
3-NP	Rat, mouse, and human primates	Systemic injections, intrastriatal injections, intraputamenal injections	Mitochondrial impairment by irreversibly inhibiting succinate dehydrogenase	Hyperkinesia, resembling early to mid-stage HD (low dose) Hypokinesia like late HD (high dose), apomorphine-induced dystonia and dyskinesia, spontaneous dyskinesia with long-term administration	Deficits in object retrieval detour task (ORDT) in nonhuman primates, radial arm water maze test of working and reference memory in rats, deficits in habituation to open-field apparatus

As shown in Table 1, the QA model effectively replicates many features of HD, including excitotoxic cell death and apoptosis. However, its acute progression contrasts with the chronicity of human HD, limiting its ability to fully capture disease dynamics. The metabolic model represented by the mitochondrial inhibitor, 3-NP, captures both the hyperkinetic and hypokinetic HD symptoms, depending on the dose and its frequency. Overall, these models are limited by the fact that they do not fully recapitulate the genetic nature of HD, the progressive nature of cell death, and the personality changes of HD patients.

### 3.2 Genetic rodent models

Genetic models better reflect the etiology and progression of HD and are central to therapeutic development. These models include transgenic, full-length, and knock-in models.

#### 3.2.1 Transgenic models

Transgenic mice (e.g., R6/2, R6/1) expressing mHTT fragments exhibit rapid disease onset, severe motor deficits, and widespread protein aggregation, making them suitable for rapid therapeutic screening (Mangiarini et al., 1996; Sun et al., 2002). However, their accelerated phenotype limits their ability to model chronic disease progression. Other fragment models (e.g., N171-82Q and N586-82Q) and transgenic rats (TgHD) offer intermediate phenotypes with extended disease courses, supporting studies on disease progression and early intervention (Ramaswamy et al., 2007; Vlamings et al., 2012; Nittari et al., 2023).

#### 3.2.2 Full-length models (YAC/BAC)

Full-length models such as YAC128 and BACHD mice express the entire mHTT protein and better recapitulate progressive neurodegeneration, motor dysfunction, and cognitive decline, making them more suitable for long-term pharmacological studies (Slow et al., 2003; Petersén et al., 2005). Key differences and similarities between these models are summarized in Table 2.

**TABLE 2:** Differences and similarities between BACHD and YAC128 models.

Feature	BACHD model	YAC128 model	Similarity	Reference
Motor degeneration	Gradual motor degeneration, with deficits in motor learning and performance	Gradual motor degeneration, including shorter rotarod fall latency	Both exhibit progressive motor degeneration	Van Raamsdonk et al. (2005), Menalled et al. (2009), Wegrzynowicz et al. (2015)
Rotarod performance	Reduced performance: balance and gait issues develop later in life		Both show decreased rotarod latency and motor deficits	Slow et al. (2003), Van Raamsdonk et al. (2005), Menalled et al. (2009), Chen et al. (2011)
Activity levels	No early hyperactivity; decreased activity from 6 months in open-field tests	Hyperactive at 3 months; hypoactive by 12 months	Both show activity impairments as the disease progresses	Slow et al. (2003), Mantovani et al. (2016)
Onset of motor deficits	Motor impairment correlated with later neuronal loss; evident by 6 months	Motor deficits begin before the hypoactive phase, with early neuronal loss	Both show motor deficits linked to neural degeneration	Lichter and Hershey (2010), Giralt et al. (2012), Mantovani et al. (2016)
Learning deficits	Impaired in motor learning, novel object recognition, sensorimotor gating, reversal learning, and strategy shifts		Both exhibit cognitive impairments, including learning deficits	Southwell et al. (2009), Aharony et al. (2015)
Neuronal dysfunction	Cortical and hippocampal dysfunction precede motor symptoms		Both models show neural network dysfunction before motor symptoms	Lichter and Hershey (2010), Giralt et al. (2012)

To enhance phenotypic expression, the YAC128 model containing 128 glutamines was created with certain specifications (Slow et al., 2003), as shown in Table 2. Compared to the original YAC models, this mouse genotype with a greater glutamine content exhibits HD-like symptoms earlier and more prominently.

#### 3.2.3 Knock-in models

Knock-in models provide the highest genetic fidelity by inserting expanded CAG repeats into the endogenous HTT locus. These models exhibit subtle, slowly progressive phenotypes, closely resembling early-stage HD (Menalled et al., 2003). However, their mild pathology and long disease course limit their utility in rapid drug screening. A comparison of knock-in models is shown in Table 3.

**TABLE 3:** Knock-in mice models (Ramaswamy et al., 2007).

Species	Construct	Promoter	CAG repeat size	Cells affected	Motor symptoms	Cognitive symptoms
HdhQ92 mouse	Replacing exon 1 of the mouse HTT gene with a mutant human exon 1	Mouse HTT promoter	92	No striatal degeneration. 4.5 M: Translocation of the mHTT protein to the nucleus	No overt symptoms	No overt symptoms
HdhQ111 mouse	Replacing exon 1 of the mouse HTT gene with a mutant human exon 1	Mouse HTT promoter	111	No striatal degeneration. 4.5 M: HTT protein translocates to the nucleus and appears punctate 24 M: Striatal gliosis	24 M: Gait abnormalities	No overt symptoms
CAG140 mouse	Inserting CAG repeats into the mouse HTT gene	Mouse HTT promoter	140	2 M: Nuclear and neuropil inclusions in the striatum, cortex, hippocampus, and cerebellum	1 M: Increase in rearing behavior using both forelimbs and open-field test locomotion 12 M: Decrease in stride length	No overt symptoms
CAG15O mouse	Inserting CAG repeats into the mouse HTT gene	Mouse HTT promoter	150	14 M: Significant increase in striatal gliosis compared to wild-type littermates. The striatum shows an increase in EM48 positive nuclear aggregates	4 M: Onset of progressive deficits on the rotarod, clasping phenotype, hypoactivity, and gait disturbances. 25 M: Significantly smaller than their wild-type littermates	No overt symptoms

As interpreted from Table 3, higher CAG repeat lengths are associated with more significant pathology, which may guide the development of future knock-in models that more closely resemble the actual disease. This notion is supported by the more severe pathology observed with CAG150 knock-in models compared to the milder pathology in CAG140 knock-in models.

#### 3.2.4 Limitations of rodent models

No single rodent model fully captures HD pathology. Transgenic models often overexpress mHTT, while toxin models lack genetic relevance. Additionally, many models fail to reproduce psychiatric symptoms and full disease progression, limiting translational predictability (Ramaswamy et al., 2007; Cardoso, 2009).

### 3.3 Large animal and non-rodent models

Large animal models offer improved translational relevance due to closer anatomical and physiological similarity to humans. Sheep models (OVT73) replicate early pathological and metabolic alterations and are valuable for longitudinal studies, although behavioral assessment remains limited (Morton, 2018; Taghian et al., 2022). Non-human primates (NHPs) exhibit complex motor and cognitive deficits and closely mimic disease progression but are constrained by ethical and cost considerations (Yin et al., 2022). Pig models provide a balance between translational relevance and practicality, showing striatal neurodegeneration and progressive phenotypes (Yan et al., 2018).

### 3.4 Cellular and alternative models

Human-induced pluripotent stem cell (iPSC)-derived neurons and brain organoids enable the study of early developmental alterations and patient-specific pathology, offering a platform for precision medicine approaches (Conforti et al., 2018; Faravelli et al., 2020). Moreover, zebrafish models are complementary model systems that provide insights into HTT function and its developmental and metabolic roles, though their relevance to adult neurodegeneration remains limited. A summary is provided in Table 4.

**TABLE 4:** Zebrafish models of Huntington’s pathology (Madivalar et al., 2024).

Method	Neuronal loss	Impaired metabolism	Motor deficits	Other phenotype
AMO knockdown	Yes	Reduced BDNF levels	Not reported	Morphological deformities. Increased mortality
AMO knockdown	Too early	Not reported	Not reported	Impaired brain development. Morphological deformities
AMO knockdown	Not reported	Increased ADAM10 activity. Increased cadherin cleavage	Not reported	Impaired brain development
AMO knockdown	Not reported	Impaired iron metabolism. Reduced hemoglobin production	Not reported	Developmental retardation and morphological deformities
4Q, 25Q, and 102Q polyQ expansion	Yes-only in 102Q	Not reported	Not reported	Morphological deformities Increased mortality
CRISPR/Cas9 deletion	No	No	Not reported	Reduced fitness and survival in adulthood

As depicted in Table 4, antisense morpholino (AMO) knockdown models consistently recapitulate early developmental abnormalities, including impaired brain development, morphological deformities, and increased mortality, suggesting their utility in modeling early pathogenic events rather than late neurodegeneration. Evidence of neuronal loss appears context-dependent (e.g., restricted to high polyQ expansions such as 102Q), indicating that zebrafish models may differentially capture toxicity thresholds associated with mHTT aggregation. Metabolic disturbances, including disrupted iron metabolism and reduced hemoglobin production, reinforce the role of non-neuronal and systemic dysfunction in HD pathogenesis. While lacking overt early phenotypes, CRISPR/Cas9-based models demonstrate reduced adult fitness and survival, suggesting their relevance for studying long-term disease progression (Madivalar et al., 2024).

### 3.5 Comparative perspective and pharmacological relevance of HD experimental models

Each experimental model serves a distinct role in HD research, particularly in relation to pharmacological applications. Toxin-induced models are primarily suited for the rapid screening of neuroprotective agents targeting excitotoxicity and mitochondrial dysfunction, whereas transgenic models are more appropriate for evaluating therapeutic efficacy in severe, early-onset phenotypes (Upadhayay et al., 2023). Full-length models, such as YAC and BAC systems, enable the assessment of disease-modifying therapies within a context of progressive pathology, while knock-in models are particularly valuable for investigating early-stage disease mechanisms and detecting subtle therapeutic effects (Kaye et al., 2021). In contrast, large animal models provide enhanced translational relevance and are increasingly used for biomarker development and validation (Rosser et al., 2022). Accordingly, the selection of an appropriate model depends on the specific therapeutic target and stage of drug development, and the combined use of multiple models is often necessary to achieve robust and predictive preclinical evaluation.

## 4 Non-neuronal pathology in HD (beyond the brain)

In addition to mHTT protein accumulation, several theories could explain the pathophysiology of neuron death in HD, including abnormal protein–protein interactions, excitotoxicity, autophagy and proteasomal dysfunction, transcriptional dysregulation, mitochondrial impairment, and oxidative stress (Kshirsagar et al., 2021; Tong et al., 2024). These mechanisms culminate in the triad of motor, cognitive, and psychiatric symptoms of HD (Kshirsagar et al., 2021; Tong et al., 2024). However, mounting evidence indicates that the HD pathology extends beyond neurons to coordinated dysfunction across diverse non-neuronal cell types such as the astrocytes, microglia, and oligodendrocytes (Ross and Tabrizi, 2011). In addition, HD is not confined to the central nervous system (CNS); rather, it is a multisystem disorder with extensive peripheral involvement, particularly the immune, gastrointestinal, cardiac, and muscular systems, contributing to disease onset and progression (van der Burg et al., 2009; Mielcarek, 2015; Rub et al., 2016). Although a large portion of research has concentrated on neuronal symptoms and mechanisms, the extent of peripheral pathology and the connections between central and peripheral systems in HD are not well understood. Understanding these peripheral contributions is crucial for developing comprehensive therapeutic approaches that address the full spectrum of HD pathophysiology.

### 4.1 Glial activation and peripheral immune system dysfunction

HD has long been recognized as a neurodegenerative disorder primarily affecting the striatum of the basal ganglia. However, mHTT is expressed not only in neurons and astrocytes but also in the brain-resident immune cells, namely, microglia. Compelling evidence has highlighted that neuroinflammation, particularly microglial activation, is involved in HD pathogenesis (Crotti and Glass, 2015). Notably, extensive microglial activation has been observed in preclinical HD as an early event, associated with striatal neurodegeneration and subclinical disease progression (Tai et al., 2007).

Microglial and astrocyte activation is considered an early and potentially exacerbating factor in the course of HD. The reactive state of these glial cells is a defining feature of neuroinflammation in the HD brain. Astrocytes and microglia typically regulate neuronal activity and maintain the optimal conditions for neuronal function. Reactive astrocytes and activated microglia contribute to HD pathogenesis by transcriptionally activating pro-inflammatory genes in response to neuron injury, thereby prolonging a chronic inflammatory state (Palpagama et al., 2019). mHTT further modifies microglial activity through several interconnected pathways, such as inflammasome activation, NF-κB signaling, and kynurenine pathway dysregulation (Abedrabbo et al., 2025). Furthermore, functional changes in energy metabolism, glutamate, and ion homeostasis occur in the reactive astrocytes (Palpagama et al., 2019). Detailed examination of postmortem tissues from HD patients has revealed a variety of astrocyte phenotypes that vary depending on the disease stage, such as reactive astrogliosis, loss of glutamate uptake, and altered lipid metabolism (Al-Dalahmah and Goldman, 2025). Furthermore, deficits in cholesterol biosynthesis in HD astrocytes have recently been shown to impair cholesterol transfer from astrocytes to neurons, thereby destabilizing synaptic signaling (Valenza, 2025). Moreover, impairment of the glymphatic system, an astrocyte-dependent mechanism for waste clearance and fluid exchange, has recently been observed in HD and contributes to the buildup of toxic proteins, including mHTT (Duan et al., 2025). As HD advances, alterations in astrocytes and microglia further intensify neuronal loss (Palpagama et al., 2019). While oligodendrocytes are often overlooked in HD pathology, recent evidence suggests that they undergo functional and transcriptional impairment in early HD, leading to reduced axonal support and myelination that may occur before severe neurodegeneration (Kamal et al., 2025). These observations highlight non-neuronal cells as dynamic and potential initiators of neurodegenerative change in HD.

Microglial activation is closely linked to systemic inflammation in HD. Microglia-derived inflammatory markers have been found to be elevated in the peripheral plasma of HD patients and mouse models and correlated with disease onset and progression (Chang et al., 2015). Since mHTT is also expressed in peripheral immune cells, the inflammatory alterations observed in peripheral plasma have been shown to mirror neuroinflammation in HD patients (Chang et al., 2015).

Peripheral immune activation is a consistent feature of HD, reflecting systemic involvement of mHTT. Elevated plasma pro-inflammatory cytokines, including tumor necrosis factor-α (TNF-α), interleukin (IL)-6, and IL-8, are detectable in HD patients (Bjorkqvist et al., 2008). Plasma IL-6 was increased even in pre-symptomatic individuals, suggesting that immune dysregulation may precede overt neurodegeneration (Bjorkqvist et al., 2008; Chang et al., 2015). However, plasma levels of the anti-inflammatory IL-4 and IL-10 increased with disease progression (Bjorkqvist et al., 2008). Plasma IL-8 was directly correlated with worsening disease symptoms and inversely with the total functional capacity (TFC) score (Bjorkqvist et al., 2008). Monocytes and macrophages exhibit a hyper-reactive phenotype with increased cytokine release upon stimulation, mirroring central microglial activation observed in HD brains (Trager et al., 2014; Crotti and Glass, 2015). mHTT expression has cell-intrinsic consequences that cause human HD myeloid cells to release excessive amounts of inflammatory cytokines. mHTT directly affects the NF-κB pathway by interacting with IKKγ, which increases IκB degradation and subsequent p65 nuclear translocation. Additionally, transcriptional changes in intracellular immunologic signaling trajectories are noted (Trager et al., 2014). Moreover, mHTT expression impairs immune cell migration via defective actin remodeling, enhances oxidative stress, and perpetuates a chronic pro-inflammatory state that may exacerbate neuronal injury via peripheral-to-central immune crosstalk (Kwan et al., 2012; Palpagama et al., 2019). Strongly impaired peripheral immune cell migration was noticed in pre-manifest HD patients, confirming that immune cells are activated in HD before symptoms arise (Kwan et al., 2012). In addition, microglial activation in the brain is thought to be partly driven by systemic inflammatory mediators, linking peripheral immune dysfunction to neuroinflammation and disease progression (Blusch and Bjorkqvist, 2025). Pro-inflammatory cytokines, chemokines, and other stimuli originating from systemic inflammation cause a leak in the blood–brain barrier (BBB), allowing for the entry of inflammatory/immune cells (Kempuraj et al., 2017; Blusch and Bjorkqvist, 2025). This expands the viewpoint that the peripheral and central immune dysfunctions work together to maintain chronic neuroinflammation in HD, paving the way for immune-based treatment approaches. In summary, both glial cell activation and peripheral immune cell dysfunction shape HD pathology, highlighting novel possibilities for HD drug development.

### 4.2 Gut–brain axis and microbiome alterations in HD progression

Recent reports highlighted the microbiota–gut–brain axis as a key modulator of neurodegenerative diseases, including HD (Loh et al., 2024; Misicka et al., 2024). High-throughput technologies revealed that diverse microorganisms within the microbiota facilitate bidirectional communication between the CNS and the enteric nervous system and between the neuroimmune and neuroendocrine systems (Liu et al., 2024). Specifically, the microbiota–gut–brain axis is considered a critical regulator of HD pathology (Wasser et al., 2020; Khoshnan, 2024; Ekwudo et al., 2025). Changes in microbial diversity and major shifts in microbial community structure have been demonstrated in HD patients and were associated with cognitive performance and clinical outcomes (Wasser et al., 2020). HD is connected to changes in the bacterial and fungal composition of the gut (Ekwudo et al., 2025). This could change microbiota-derived metabolites, such as bile acids, short-chain fatty acids, and branched-chain amino acids, which mediate communication between the gut and brain. Disruption in this communication could trigger neuroinflammatory responses (Ekwudo et al., 2025).

In HD, most cell lineages express mHTT, and its toxicity in multiple organs exacerbates the neurological and cognitive symptoms. HD patients suffer from esophageal and gastric inflammation, chronic diarrhea, and constipation and are susceptible to diabetes. mHTT expression in enteric neurons and intestinal epithelial cells contributes to gastrointestinal dysmotility and barrier dysfunction (van der Burg et al., 2011). Gut microbiota dysbiosis—characterized by altered abundance of bacterial taxa such as *Bacteroides*, Akkermansia, and Lactobacillus—has been documented in both human and animal HD models, in conjunction with mHTT aggregation and abnormal behavior (Wasser et al., 2020; Khoshnan, 2024; Ekwudo et al., 2025). These microbial shifts can influence systemic inflammation, immune activation, and neurotransmitter metabolism, thereby indirectly affecting CNS function through a microbiota–gut–immune–CNS trajectory (Strandwitz, 2018). Furthermore, gut microbiota-derived metabolites and endotoxins may promote neuroinflammatory signaling and modulate microbial activity, reinforcing the concept that intestinal homeostasis plays an impeccable role in HD progression (Du et al., 2020). The therapeutic modulation of the microbiota, through probiotics or dietary interventions, has thus emerged as a potential adjunct strategy to mitigate HD pathology and other neurodegenerative diseases (Wasser et al., 2020; Loh et al., 2024; Misicka et al., 2024). Nevertheless, additional translational and longitudinal research is required to further elucidate this emerging area, and clear recommendations for future research are needed to deepen our understanding of the connections and associated networks between gut microbiota and HD etiology and pathogenesis.

### 4.3 Cardiac and skeletal muscle degeneration in HD patients

Beyond the nervous system, ample evidence underscores a widespread, multiorgan pathology involving the heart and skeletal muscles. HD leads to significant peripheral tissue degeneration in these organs, in conjunction with mHTT’s ubiquitous expression and toxic effects. For HD patients, early-onset cardiovascular disease is the second most common cause of mortality (Mihm et al., 2007; Melkani, 2016). HD induces cardiac amyloidosis and other cardiovascular diseases (Melkani, 2016). Cardiomyopathy is a recognized but underappreciated manifestation of HD, with reports of conduction abnormalities, reduced contractility, and heart failure contributing to premature mortality (Mihm et al., 2007; Melkani, 2016; Critchley et al., 2018). Several mechanisms are involved in HD-induced cardiomyopathy, including autophagic defects, protein misfolding, mitochondrial dysfunction, oxidative stress, and cellular death (Melkani, 2016). In HD transgenic mice, mHTT expression had potential cardiotoxic effects, with changes in mitochondrial ultrastructure and increased cardiac protein nitration and lysine acetylation, which were associated with impaired cardiac performance (Mihm et al., 2007). mHTT aggregates in cardiomyocytes disrupt mitochondrial function, calcium homeostasis, and autophagic flux, leading to energetic deficits and cell death (Melkani, 2016). mHTT-independent HD-induced cardiomyopathy has also been found in transgenic HD mouse models. In these models, cardiac MRI revealed significant functional alterations in symptomatic mice, including contractile failure secondary to dilated cardiomyopathy. Alongside this, apoptotic cardiomyocyte loss, re-expression of fetal genes, and interstitial fibrosis were observed but without HD-specific transcriptional dysregulation or mHTT aggregates in cardiac tissue, suggesting altered central autonomic pathways as an alternative mechanism (Mielcarek et al., 2014). Together, these findings could offer potential targets for new disease-modifying treatments.

Similarly, wasting of the skeletal muscles is a hallmark of HD (Zielonka et al., 2014). Muscle atrophy caused by myofiber degeneration and muscle fibrosis is the major pathological feature of genetic muscle diseases. In addition, body weight loss, muscle weakness, fatigue, and contractile abnormalities have been observed. The skeletal muscles exhibit atrophy, fiber-type switching, and mitochondrial abnormalities even before clinical motor symptoms appear (Zielonka et al., 2014). HD triggered a characteristic gene expression profile in the skeletal muscle of HD patients and mouse models that mirrors the start of a transition from fast to slow-twitch muscle fiber types (Strand et al., 2005). Formation of polyglutamine inclusions in the skeletal muscles was observed in an HD mouse model (Sathasivam et al., 1999). The muscle pathology reflects a systemic metabolic impairment driven by mHTT-induced transcriptional dysregulation of genes involved in ATP production and branched-chain amino acid metabolism, as well as chronic inflammation and oxidative stress (Krzyszton-Russjan et al., 2013). Other mHTT-independent mechanisms have been noted, such as increased myostatin/activin A signaling in HD transgenic models; inhibition of this axis has been shown to increase muscle mass (Bondulich et al., 2017). These peripheral changes not only affect the energy balance and the physical performance of HD patients but also mirror the neurodegenerative mechanisms occurring in the brain, offering potential drug targets.

Altogether, HD is not just a neuronal disease but is considered a systems-level disorder that involves coordinated dysfunction across non-neuronal brain cells, immune cells, gut microbiota, the heart, and skeletal muscles, as summarized in Table 5. Peripheral pathologies often precede or parallel central neurodegeneration. By redefining HD in this context, this review adds new peripheral therapeutic targets and enhances our mechanistic comprehension of HD. Targeting systemic inflammation, gut microbiota, and metabolic tissues may offer complementary therapeutic avenues along with CNS-directed interventions.

**TABLE 5:** Non-neuronal pathology in HD.

System	Main pathological feature	Mechanistic insight	References
Glial activation and peripheral immune system dysfunction	Neuroinflammation Microglial activation	mHTT expression in microglia activates NF-κB signaling and the inflammasome and dysregulates the kynurenine pathway	Crotti and Glass (2015), Palpagama et al. (2019), Abedrabbo et al. (2025)
	Astrocyte activation	Reactive astrogliosis, glutamate uptake loss, and altered lipid metabolism and ion homeostasis, along with cholesterol biosynthesis deficits and impaired glymphatic system	Al-Dalahmah and Goldman (2025), Duan et al. (2025), Valenza (2025)
	Reduced axonal support and myelination	Oligodendrocyte functional impairment	Kamal et al. (2025)
	Elevated plasma levels of IL-6, TNF-α, and IL-8 in HD patients, while increased levels of IL-4 and IL-10 are correlated with disease progression Plasma IL-8 inversely correlates with the TFC score Elevated plasma IL-6 in pre-manifest individuals Hyper-reactive monocytes and macrophages Chronic systemic inflammation	mHTT expression in immune cells activates NF-κB and oxidative pathways, stimulates their excessive production of cytokines, and impairs immune cell migration	Bjorkqvist et al. (2008), Kwan et al. (2012), Trager et al. (2014), Chang et al. (2015)
		Peripheral cytokines cross-talk with CNS microglia, promoting neuroinflammation Immune activation correlates with disease burden	Kwan et al. (2012), Palpagama et al. (2019)
Gut-brain axis and microbiome alterations	Gastrointestinal dysmotility and barrier dysfunction Altered gut microbiota diversity and composition in HD patients in correlation with cognitive performance and clinical outcomes Alterations in gut bacterial and fungal composition Changes in microbiota-derived metabolites Increased gut inflammation	mHTT expression in enteric neurons and epithelial cells	van der Burg et al. (2011)
		Microbiota dysbiosis is associated with mHTT aggregation and abnormal behavior	Wasser et al. (2020), Ekwudo et al. (2025)
		Dysbiosis drives systemic inflammation and modulates neurotransmitter metabolism	Strandwitz (2018)
		Microbial metabolites and endotoxins influence neuroinflammatory signaling and microbial activity	Du et al. (2020)
Cardiac pathology	Cardiomyopathy, conduction abnormalities, reduced contractility Cardiac amyloidosis Cardiac failure contributing to early mortality	mHTT has potential cardiotoxic effects, with increased cardiac protein nitration and lysine acetylation and changes in mitochondrial ultrastructure	Mihm et al. (2007)
		mHTT aggregates in cardiomyocytes disrupt mitochondria, calcium homeostasis, and autophagy and cause oxidative stress and energetic and metabolic impairment, leading to cell death	Melkani (2016)
		mHTT-independent effects, including a contractile failure, apoptotic cardiomyocyte loss, fetal genes’ re-expression, and interstitial fibrosis caused by altered central autonomic pathways	Mielcarek et al. (2014)
Skeletal muscle atrophy	Muscle wasting, early-onset muscle atrophy, and fiber-type switching Reduced mitochondrial content and oxidative capacity Energy metabolism deficits	Formation of polyglutamine inclusions	Sathasivam et al. (1999)
		mHTT alters transcription of metabolic and myogenic genes; mHTT causes mitochondrial dysfunction, oxidative stress, and inflammation parallel to CNS pathology	Strand et al. (2005), Krzyszton-Russjan et al. (2013)
		mHTT-independent mechanism such as increased myostatin/activin A signaling	Bondulich et al. (2017)

CNS, central nervous system; HD, Huntington’s disease; IL, interleukin; mHTT, mutant huntingtin; NK-κB, nuclear factor-κB; TFC, total functional capacity; TNF-α, tumor necrosis factor-α.

## 5 Lifestyle and environmental factors

HD is influenced by lifestyle and environmental factors, with striking gene-environment interaction features (Hannan, 2022). HD was initially viewed as a disease governed solely by genetics; however, this perspective was changed by the pioneer study of van Dellen et al. (2000), which showed that environmental enrichment (enhanced sensory stimulation, cognitive activity, and physical exercise) delayed disease onset in transgenic HD mice. Clinical translation through observational studies in HD patients has supported that variations in age at onset and progression rate are influenced by lifestyle and environmental modifications (Wexler et al., 2004; Trembath et al., 2010). While age at onset is negatively correlated with CAG repeat length, CAG repeats account for only 50%–70% of the variability in age at onset (Trembath et al., 2010), supporting the potential role of epigenetic and environmental factors in delaying disease onset or progression. Further human and preclinical studies confirmed that HD is modulated by environmental enrichment, mental and physical activity, stress, and diet (Novati et al., 2022).

Lifestyle interventions, including stress management, cognitive enrichment, physical activity, and a healthy diet, have been shown to delay the age at onset or the progression of HD (Novati et al., 2022). Physical activity was associated with improved cognitive performance in prodromal/early HD patients (Wallace et al., 2016). In HD patients, an active lifestyle, regular physical exercise, and behavioral therapies resulted in beneficial effects on HD symptoms (Novati et al., 2022). Adherence to the Mediterranean diet is associated with reduced motor and cognitive impairment, and omega-3 fatty acid-based treatments improve motor function in HD patients (Christodoulou et al., 2020). A recent national-based study highlighted that high caffeine consumption was associated with reduced mortality of HD (Cubo et al., 2025). In pre-manifest HD individuals, modifiable factors such as education, abstinence from smoking, low-to-moderate alcohol use, and a healthy body mass index were connected to a slower progression rate (Gil-Salcedo et al., 2024).

Another key lifestyle factor in HD-related gene–environment interactions is intellectual enrichment (Papoutsi et al., 2022). In a longitudinal, international, multicenter study (TRACK-HD) by Papoutsi et al. (2022), intellectual enrichment (defined as higher education, verbal intelligence, and intellectually engaging occupation) was associated with delayed HD onset and superior overall cognitive function. Additionally, intellectual enrichment interacts with genetic factors through the Val66Met polymorphism in the brain-derived neurotrophic factor (BDNF) gene (Papoutsi et al., 2022), suggesting a highly intriguing connection to BDNF. Both the HD mutation and relevant environmental factors (such as physical exercise and cognitive enrichment) alter BDNF expression (Pang et al., 2006). MutS homolog3 (MSH3) is a DNA mismatch repair gene that significantly impacts cognitive function and neurodegeneration in the cerebral cortex, consistent with its role as a genetic modulator of HD by influencing somatic CAG-repeat stability (Papoutsi et al., 2022).

With this information, novel preventive and treatment strategies, such as enviromimetics, could be developed to delay onset and improve outcomes in brain disorders, including HD (McOmish and Hannan, 2007). Therapeutics known as “enviromimetics” imitate or enhance the beneficial effects of environmental stimulation, such as cognitive stimulation and physical exercise (McOmish and Hannan, 2007). The therapeutic effects of exercise mimetics, a subclass of enviromimetics, have been highlighted in a recent study, paving the way for harnessing the impact of physical activity in HD management (Gubert and Hannan, 2021). One important mechanism of enviromimetics is targeting BDNF, its related receptors, and downstream signaling pathways (McOmish and Hannan, 2007; Papoutsi et al., 2022). Other potential mechanisms of environmental interventions include increased adult neurogenesis, trophic support, synaptic plasticity, and other types of experience-dependent cellular plasticity (Mo et al., 2015). Notably, environmental enrichment showed an antidepressant-like effect, reduced monoamine levels, particularly in the striatum, enhanced hippocampal neuroplasticity, improved motor performance, and delayed motor deficit progression in HD mice (Placido et al., 2023).

Although there is no curative treatment for HD, lifestyle therapies may serve as useful adjuvants as they are accessible, low-cost, and generally safe. Combining environmental, dietary, and lifestyle modifications can not only delay disease onset but also help control symptoms and improve quality of life, particularly when implemented in a multifaceted manner with professional support, including speech therapy, mental health services, and physical therapy. These environmental and lifestyle factors influence disease progression and patient response, underscoring the importance of adaptable and personalized therapeutic strategies.

## 6 Sex differences in HD progression and treatment response

Growing evidence indicates that sex-related variations can affect multiple pathological dimensions in HD, including its clinical course, age of onset, progression rate, and response to treatment (Zielonka and Stawinska-Witoszynska, 2020). These variations appear to originate from complex interactions involving hormonal influences, genetic background, and sex-specific immune and metabolic profiles (Shepherd et al., 2021). Considering sex as a biological variable in HD is, therefore, vital for understanding HD heterogeneity and designing more tailored therapeutic strategies.

### 6.1 Hormonal influences

Sex hormones, particularly estrogens, are believed to influence HD pathology via both neuroprotective and neurotrophic mechanisms. Studies using experimental HD mouse models such as R6/1 and YAC128 have shown that 17β-estradiol treatment decreases mHTT aggregation, preserves striatal neurons, and consequently improves motor performance. Such protective effects were linked to enhanced BDNF expression, mitochondrial stabilization, and reduced oxidative stress (Kreilaus et al., 2016; Upadhayay et al., 2023). In contrast, estrogen depletion, either via ovariectomy or due to age-related decline, has been associated with accelerated neurodegeneration in HD models, emphasizing the importance of estrogen signaling in maintaining neuronal integrity (Anukulthanakorn et al., 2013; Cheng et al., 2021).

### 6.2 Variability in symptom onset and severity between male and female individuals

Analyses of large HD registries have reported notable sex-related differences in HD course. In general, female patients often present with earlier onset of motor manifestations and more rapid decline in functional capacity, while male HD patients possess higher rates of psychiatric and cognitive deteriorations (Hentosh et al., 2021; Stanisławska-Sachadyn et al., 2024; Yao et al., 2024). Additionally, depressive symptoms are believed to be more frequent and pronounced among female HD patients, which can be possibly attributed to hormonal fluctuations and the ability of estrogen to modulate serotonergic pathways (Hentosh et al., 2021). On the other hand, elevated levels of irritability and aggression were frequently observed in male HD patients (Fisher et al., 2014).

Collectively, these findings underscore that sex-specific mechanisms contribute to the heterogeneity of HD manifestations and should be considered when assessing disease progression and treatment efficacy, further highlighting the importance of tailored drug delivery approaches to optimize therapeutic outcomes.

## 7 Challenges in HD diagnosis

Diagnosing HD involves unique clinical and ethical complexities that pose significant challenges for clinicians, patients, and families. Diagnostic difficulties stem from the variability in clinical presentation, limitations of existing diagnostic criteria, ethical considerations related to genetic testing, and broader implications of disease awareness and disclosure.

The clinical diagnosis of manifest HD (mHD) traditionally relies on recognizing a combination of motor (particularly chorea) and non-motor (cognitive and psychiatric) symptoms, alongside genetic testing that identifies expanded CAG repeats in the HTT gene. A confirmed diagnosis requires both clinical evidence and molecular genetic confirmation, with 36 or more CAG repeats generally considered diagnostic (Margolis and Ross, 2003; Mian et al., 2024).

However, the diagnosis is not always straightforward. HD has a highly variable phenotype; symptoms may not initially include the classic motor features, especially early in the disease—cognitive and psychiatric manifestations such as depression, apathy, and psychosis may precede chorea by years. These non-motor symptoms are often non-specific and overlap with common psychiatric disorders, leading to misdiagnosis or delayed recognition. Moreover, early motor signs may be subtle and escape notice by patients and clinicians, particularly when patients have poor awareness of their symptoms (McCusker and Loy, 2017).​

Imaging techniques such as MRI can show brain changes such as caudate atrophy, supporting diagnosis, but these findings are not pathognomonic and can overlap with other neurodegenerative diseases. Furthermore, some patients may have normal imaging findings early in the disease. The lack of a precise clinical threshold to define disease onset means that neurologists must rely on diagnostic confidence scales (e.g., Unified Huntington Disease Rating Scale Diagnostic Confidence Limits), which are somewhat subjective and depend on examiner expertise (Mian et al., 2024).

The identification of the HD mutation decades ago introduced new diagnostic paradigms with predictive genetic testing for at-risk individuals before symptom onset. A person carrying the pathogenic CAG expansion can receive a “genetic diagnosis” years before clinical manifestations appear. This pre-manifest diagnosis raises ethical dilemmas concerning if, when, and how to disclose the diagnosis to asymptomatic individuals.

Disclosure decisions must balance the person’s right to know with their right not to know, avoiding potential psychological harm or discrimination. Some may prefer early knowledge for life planning and participation in clinical trials. Others may choose to remain unaware to maintain psychological wellbeing and avoid stigma. The timing of clinical diagnosis disclosure is further complicated by variable disease progression rates and phenotypic variability, making predictions about onset uncertain.​

Adding to the complexity, early manifestations of HD, especially psychiatric symptoms, may be misattributed to other causes, leading to delayed recognition or inappropriate treatment. Disparities also exist; studies document later diagnosis in certain ethnic groups and in patients presenting with mainly psychiatric symptoms, reflecting challenges in healthcare access and disease awareness. Male patients and individuals with higher educational attainment have also been found to face delays in diagnosis, suggesting socioeconomic and cognitive biases in clinical recognition.

Several rare genetic and acquired conditions can clinically mimic HD, and a negative genetic test does not exclude another disorder. Thus, a broad differential diagnosis is important, especially in patients without a family history or with atypical presentations. The presence of juvenile or late-onset cases further complicates diagnosis as these may not present with typical chorea or cognitive impairment patterns.

Additionally, there is currently no reliable biomarker or neuroimaging modality to definitively identify disease onset before motor symptoms arise, limiting early intervention and clinical trial enrollment. Ongoing research aims to identify molecular markers, including measurements of mutant huntingtin in cerebrospinal fluid, but such tools remain experimental.

## 8 Repurposed drug therapies

Several repurposed drug therapies have been explored for the management of HD (Table 6), aiming to improve symptoms or to modify disease progression (Tabrizi et al., 2022). Drug repurposing approaches offer several practical advantages because these compounds have well-established safety profiles and pharmacokinetic data, which can reduce the cost and time needed for their clinical development and approval as HD therapies (Clout et al., 2019).

**TABLE 6:** Investigational repurposed drugs for HD.

Agent	Pharmacological class	Rationale in HD	Evidence and trial status	Clinical challenges
Pridopidine	Dopamine D_2_ receptor stabilizer (Sahlholm et al., 2018) + Sigma-1 receptor agonist (Naia et al., 2021)	Improves neuronal health, stabilizes neuronal networks, and helps motor symptoms (Eddings et al., 2019; Naia et al., 2021)	In 1,119 patients across four RCTs, it improved the unified HD rating scale (UHDRS)-modified motor score (mMS) (Chen et al., 2021)	Higher doses (≥90 mg/day) increased total adverse events such as nasopharyngitis and insomnia (Chen et al., 2021)
Cysteamine bitartrate	Cystine-depleting agent used in cystinosis (Medic et al., 2017)	Inhibits transglutaminase and increases BDNF (Borrell-Pagès et al., 2006)	No statistically different effects were shown in 96 patients with early-stage HD (Verny et al., 2017) Preclinical animal models show improvements (Abd El-Gawad et al., 2009)	The dose-limiting adverse effects included motor dysfunctions and nausea (Dubinsky and Gray, 2006)
Lithium citrate	Mood stabilizer used in bipolar disorders (Jope, 1999)	Promotes neuronal survival (Senatorov et al., 2004) and increasing neurotrophic factors (Chuang, 2004)	In a case series of three patients, lithium delayed the occurrence of chorea and stabilized the mood (Danivas et al., 2013). Preclinical animal models show improvements (Vazey and Connor, 2010; Pouladi et al., 2012)	Narrow therapeutic window (Waring, 2006)
Nilotinib	Tyrosine kinase inhibitor (Tian et al., 2018)	Promotes autophagy and clearance of mutant proteins (Anderson et al., 2022)	Small, early, and explorative (Phase Ib) study on 10 HD patients (Anderson et al., 2022)	Safety and tolerability need more evaluation
SOM3355	β1-adrenoceptor antagonist (Frishman et al., 1988) + VMAT2 inhibitory activity (Gamez et al., 2023)	Reduces choreic movements such as other VMAT2 inhibitors but perhaps with fewer side effects (Gamez et al., 2023)	In Phase 2a trial, improved total maximal chorea (TMC) score of UHDRS were observed(Gamez et al., 2023)	Well tolerated with mild or moderate adverse events, usually related to cardiovascular system (Gamez et al., 2023)
Metformin	Biguanide used for hyperglycemia (Campbell et al., 1996) + AMPK activator (Agius et al., 2020)	Promotes autophagy, reduces mHTT aggregation, enhances mitochondrial biogenesis (Jin et al., 2016)	Involved in an ongoing clinical study, TEMET-HD including 60 patients (Maeso et al., 2024) Improves motor function and reduces aggregates in HD mice (Arnoux et al., 2018; Sanchis et al., 2019)	—
Rapamycin (sirolimus)	Macrocyclic immunosuppressant + mTOR inhibitor (Wang and Eisen, 2022)	Activates autophagy, clears mHTT (Sarkar et al., 2009)	Improves behavior in HD experimental models (Tsai et al., 2013; Roth et al., 2023)	—
Roflumilast	Phosphodiesterase-4 inhibitor (PDE-4) used for the treatment of chronic obstructive pulmonary disease (Taha et al., 2026)	Inhibits PDE-4 and augments the cAMP/CREB/BDNF axis Mitigates pyroptosis, ferroptosis, and glial activation	Improves functional and motor behavior in the 3-NP rat model of HD (Taha et al., 2026)	—

The first successful example of a repurposed drug for HD is tetrabenazine, a vesicular monoamine transporter type 2 (VMAT2) inhibitor that was originally intended for hyperkinetic movement disorders (Frank, 2010). Its deuterated derivative, deutetrabenazine, was subsequently developed *de novo* in order to improve pharmacokinetic stability and tolerability and was later approved specifically for HD chorea (Tripathi et al., 2024). Both agents act by decreasing excessive dopamine in the synaptic cleft, thereby controlling involuntary movements (Jankovic, 2016).

Later on, several other repurposed compounds were recognized for their neuroprotective or disease-modifying potential (Pérez-Arancibia et al., 2023). Pridopidine, a sigma-1 receptor agonist that was initially developed as a dopaminergic stabilizer, has demonstrated modest improvements in motor function in clinical trials (Drewes et al., 2025). In addition, cysteamine bitartrate, originally indicated for cystinosis, demonstrates antioxidant and neurotrophic effects in preclinical reports; however, its clinical utility in HD needs further reports (Borrell-Pagès et al., 2006). Lithium citrate, a well-known mood stabilizer, has also attracted interest due to its ability to modulate intracellular signaling pathways and support neurotrophic factor expression; however, its narrow therapeutic index limits its clinical use in HD (Wei et al., 2001; Pouladi et al., 2012).

Nilotinib, a tyrosine kinase inhibitor approved for leukemia, is being investigated for its ability to promote autophagy and enhance the clearance of mHTT protein aggregates (Anderson et al., 2022). Similarly, bevantolol hydrochloride (SOM3355), which was originally developed for the treatment of hypertension, is being evaluated in phase 2 trials for its potential to reduce choreic movements through dual β1-adrenergic blockade and VMAT2 inhibition (Gamez et al., 2023). Despite these advances, most repurposed therapies remain symptomatic rather than disease-modifying, and their long-term safety and efficacy require further confirmation in larger, controlled studies (Tabrizi et al., 2022).

Moreover, metformin, a first-line antidiabetic agent, is being repurposed for its potential neuroprotective effects in HD, owing to its ability to act as an AMP-activated protein kinase (AMPK) activator (Agius et al., 2020). In experimental HD models, metformin has been reported to enhance the autophagic clearance of neuronal deposits. In addition, metformin can improve mitochondrial activity, ameliorate oxidative stress conditions, and upregulate BDNF expression, which collectively protect striatal neuronal structures (Jin et al., 2016; Arnoux et al., 2018; Sanchis et al., 2019; Agius et al., 2020). Clinical observations in HD patients also suggested a possible association between metformin use and slower disease progression, although the data remain preliminary (Maeso et al., 2024).

Rapamycin, originally approved as an immunosuppressant for preventing organ transplant rejection, has also been considered for repurposing in HD due to its potent mTOR-inhibitory effects. In HD models, rapamycin has been reported to increase autophagic clearance of mHTT aggregates, reduce neuronal toxicity, and consequently improve motor function in transgenic mice (Sarkar et al., 2009; Tsai et al., 2013; Roth et al., 2023). Finally, repurposing the phosphodiesterase-4 inhibitor, roflumilast was suggested for managing HD, based on an experimental study in a 3-NP-induced rat model of HD-like neurodegeneration. This study showed that roflumilast mitigated pyroptotic and ferroptotic neuronal cell death pathways, obliterated microglial activation, and enhanced synaptic plasticity, leading to motor improvement (Taha et al., 2026).

## 9 Neurophysiological barriers impeding CNS drug delivery in HD

Delivering therapeutic molecules to the CNS remains one of the most formidable challenges in treating HD. The central issue lies in overcoming the highly selective physiological barriers that shield the brain, particularly the BBB and the blood–cerebrospinal fluid barrier (BCSFB), which together strictly regulate molecular movement between the systemic circulation and neural tissue (Abbott et al., 2010; Ahmed et al., 2026). Understanding these barriers in the context of HD pathophysiology, along with patient-specific factors such as disease stage, systemic inflammation, and sex differences, is crucial for designing effective CNS-targeted drug delivery strategies.

### 9.1 Blood–brain barrier

The BBB consists of a dense network of brain capillary endothelial cells interconnected by tight and adherent junctions, supported by pericytes and astrocytic end-feet (Khan et al., 2025). This structure serves as a crucial protective interface, maintaining the brain’s microenvironment and preventing harmful agents, toxins, and pathogens from entering (Upadhyay, 2014). Although this selectivity preserves neural function, it simultaneously restricts the penetration of many therapeutic agents, including small molecules, peptides, proteins, and nucleic acids (Sayed et al., 2021; Sayyed et al., 2026). Drug physicochemical properties, such as lipophilicity, molecular weight, and charge, further dictate BBB permeability, highlighting the importance of rational formulation design to enhance CNS bioavailability.

Efforts to facilitate drug delivery across the BBB often face complications such as limited receptor availability, competition with endogenous ligands, and receptor saturation, which together reduce the fraction of the drug that successfully reaches the target site. Some studies have explored transient BBB disruption using osmotic or chemical agents to enhance permeability (Burgess et al., 2015). However, such interventions compromise barrier integrity, allowing unwanted substances to infiltrate the brain and causing potential neurotoxicity or homeostatic imbalance (Ahlawat et al., 2020). Consequently, nanotechnology-based carriers, including liposomes, polymeric nanoparticles, and receptor-targeted systems have gained prominence as safer and more controllable alternatives capable of enhancing drug penetration across the BBB while minimizing systemic toxicity (Elmahboub et al., 2025).

### 9.2 Blood–cerebrospinal fluid barrier

While the BBB presents the primary obstacle to CNS drug delivery, additional barriers such as the BCSFB further restrict therapeutic access, highlighting the need for multi-targeted delivery strategies. The BCSFB represents an additional checkpoint for CNS drug delivery, formed primarily by the epithelial cells of the choroid plexus (CP) (Solar et al., 2020). These cells are tightly linked by junctional complexes, producing a highly regulated interface that separates blood from cerebrospinal fluid (CSF). The BCSFB also contains an array of efflux transporters and detoxifying enzymes, which collectively restrict the entry of xenobiotic and metabolite into the CNS (Ueno et al., 2016). Formulation strategies that exploit receptor-mediated transport at the CP or transient modulation of efflux transporters could improve CSF drug concentrations, offering an alternative or complementary route to BBB-targeted delivery. Given its strategic location and regulatory mechanisms, researchers have explored drug delivery strategies targeting the choroid plexus epithelium. However, most conventional approaches still focus on the microvascular endothelium of the brain (Pardridge, 2016). Together with transporter systems and efflux mechanisms, these barriers define the key physiological constraints that must be overcome for successful HD therapy.

### 9.3 Transporter systems and efflux mechanisms

ATP-binding cassette (ABC) transporters are central to maintaining the brain’s chemical homeostasis (Girardin, 2006). Members of this family, such as multidrug resistance protein (MDR1), ATP-binding cassette subfamily C member 4 (ABCC4), and breast cancer resistance protein (BCRP), actively expel a variety of substrates, including therapeutic compounds, from endothelial cells back into the bloodstream (Alyautdin et al., 2014). These transporters contribute to synaptic ionic balance by regulating levels of potassium, sodium, and calcium (Cuomo et al., 2015). Although several molecular transport routes have been characterized, including simple diffusion, transcellular transport, receptor-mediated transcytosis (RMT), adsorptive-mediated transcytosis (AMT), carrier-mediated transport, and cell-mediated delivery (Khan et al., 2018), each presents inherent limitations. For instance, receptor- or carrier-mediated systems may allow precise targeting, yet they often suffer from low drug-loading capacity or premature degradation. Advanced nanoparticle design, surface functionalization with ligands, or dual-targeting strategies can overcome these limitations by enhancing CNS accumulation and prolonging drug residence time. For example, transcellular pathways often suffer from low drug-loading capacity and premature cargo release (Zhang et al., 2023). Carrier-mediated systems are restricted to compounds structurally resembling endogenous substrates (Arvanitis et al., 2020). Furthermore, in receptor-mediated endocytosis, a substantial proportion of vesicles undergo endosomal degradation, reducing effective transport to the brain (Cabezon et al., 2017). Table 7 summarizes neurophysiological barriers in HD.

**TABLE 7:** Summary of major physiological barriers and their impact on drug delivery in HD.

Barrier/System	Structural component	Physiological role	Key challenges for drug delivery	Ref.
Blood–brain barrier (BBB)	Brain capillary endothelial cells, tight/adherent junctions, pericytes, astrocytic end-feet	Maintains brain structure and prevents entry of toxins and pathogens	Restrictive permeability; receptor saturation; limited transport; neurotoxicity risk upon disruption	Upadhyay (2014), Sayed et al. (2021), Khan et al. (2025)
Blood–cerebrospinal fluid barrier (BCSFB)	Choroid plexus epithelial cells with tight junctions, efflux transporters, detoxifying enzymes	Separates blood from CSF; regulates solute exchange	Efflux of xenobiotic and drugs; enzymatic degradation; limited targeting strategies	Pardridge (2016), Ueno et al. (2016), Solar et al. (2020)
Transporter systems and efflux mechanisms	ATP-binding cassette (ABC) transporters (MDR1, ABCC4, and BCRP)	Maintain ionic and metabolic homeostasis; regulate synaptic ion balance	Active drug efflux; low loading capacity; endosomal degradation; substrate specificity	Alyautdin et al. (2014), Cuomo et al. (2015), Khan et al. (2018)

### 9.4 Translational barriers in HD drug development

Despite substantial advances in understanding HD pathophysiology and the development of innovative therapeutic strategies, successful clinical translation remains limited. Several key barriers impede the progression from preclinical findings to effective clinical therapies (Rosser et al., 2022).

First, the poor predictive value of experimental models presents a major limitation as many animal models fail to fully recapitulate the complexity and progressive nature of human HD (Han et al., 2024). Second, neurophysiological barriers, particularly the BBB, significantly restrict drug penetration and CNS bioavailability (Sayed et al., 2021). Third, pharmacokinetic issues, off-target effects, and long-term safety further complicate therapeutic development (Ferguson et al., 2022).

From a formulation perspective, challenges such as drug instability, limited solubility, and inefficient targeted delivery reduce therapeutic efficacy. Nanotechnology-based systems offer promising solutions, yet their scalability, reproducibility, regulatory approval, and long-term safety remain critical concerns. Altogether, these translational barriers underscore the need for integrative approaches that combine disease biology, advanced drug delivery strategies, and clinically relevant models to accelerate the development of effective HD therapies. These challenges, as summarized in Figure 1, link HD pathophysiology to therapeutic solutions.

## 10 Evolution of nanosystems for brain-targeted drug delivery

Over the past 2 decades, the integration of nanotechnology into medicine has revolutionized strategies for CNS drug delivery. This transformation was primarily driven by the persistent challenge of transporting therapeutic molecules across the brain’s formidable biological barriers, particularly the BBB and brain immune defense systems (Albash et al., 2025; Farag et al., 2025). In HD, these barriers are further complicated by neuroinflammation, altered BBB permeability, and disease-specific transporter dysregulation, which collectively limit drug access to affected striatal and cortical regions.

Traditional small-molecule and lipophilic drugs frequently fail to reach therapeutic concentrations in the brain because of inadequate permeability through the BBB, limited interaction with transport systems, and rapid systemic clearance (Tawfik et al., 2023). Moreover, repeated systemic administration often triggers peripheral toxicity or immune responses, further restricting the utility of conventional formulations. The tight junctions between endothelial cells, specialized efflux transporters, and the highly selective permeability of the BBB further restrict the passage of most exogenous compounds (Upadhyay et al., 2024).

To overcome these limitations, nano-therapeutics have emerged as an innovative and effective class of delivery systems. Owing to their nano-metric dimensions and tunable physicochemical properties, nanocarriers can efficiently navigate the narrow brain capillaries and cross endothelial barriers via endocytosis or transcytosis mechanisms (Danaei et al., 2018). These systems can also bypass P-glycoprotein (P-gp) and other efflux transporters, enhancing CNS drug retention. The selection of an appropriate nanocarrier depends on several factors, including particle size, shape, surface charge, composition, and biocompatibility, as well as formulation stability and production cost (Elsharkawy et al., 2023; Ahmed et al., 2025b; Fahmy et al., 2026). Optimized design enables enhanced pharmacokinetic profiles, site-specific delivery, and reduced systemic toxicity, ultimately improving patient safety and compliance (Ahmed Tawfik et al., 2023; Eldeeb et al., 2026). Furthermore, recent developments in ligand-directed and receptor-mediated nanocarriers have paved the way for more selective drug delivery approaches. Decorating nanoparticles with BBB-targeting moieties such as peptides, antibodies, aptamers, or transporter ligands enhances receptor interaction and facilitates transcytosis into neuronal tissues (Ashique et al., 2021). By tailoring physicochemical characteristics and surface functionalities, these nanosystems provide a controlled and sustained drug release profile within the brain microenvironment. Overall, nanosystems represent a paradigm shift in the treatment of neurodegenerative disorders, including HD, offering a pathway toward precise, efficient, and minimally invasive CNS drug delivery. Integration of disease-specific targeting, combination therapy potential, and adaptive release mechanisms positions nanocarriers as the next-generation platform for personalized HD therapeutics.

### 10.1 Innovative nano-technological strategies to overcome BBB

Several strategic approaches have been developed to enable nanoparticles to overcome the BBB and optimize CNS drug bioavailability. These include passive targeting, physical or invasive techniques, cell-penetrating peptides (CPPs), active targeting, and intranasal delivery. The following subsections highlight these major strategies and their mechanisms.

#### 10.1.1 Passive targeting

Passive targeting relies on energy-independent transport mechanisms, such as simple diffusion and paracellular or transcellular movement, to facilitate drug delivery across biological membranes (Sharma et al., 2019). It utilizes pathophysiological differences between healthy and diseased tissues, particularly the enhanced permeability and retention (EPR) effect, to promote selective drug accumulation in sites such as brain tumors or inflamed tissues (Wen and Meng, 2014). Nanocarriers, including liposomes, emulsions, and nanoparticles within the 10–100 nm size range and with hydrophilic surfaces, can evade rapid clearance by the reticuloendothelial system, allowing for longer systemic circulation and improved interaction with target tissues (Wu, 2021). In HD, reactive astrocytes and microglial activation may locally enhance BBB permeability, creating microenvironments where passive targeting could be more effective. For example, gold nanoparticles (AuNPs) coated with polyethylene glycol (PEG) or TAT peptides can passively cross the BBB due to their small size and endothelial affinity (Cheng et al., 2014). Their ultra-small size (0.9–1.5 nm) is comparable to ion channels such as Na^+^, K^+^, and Ca^2+^ channels, potentially facilitating passage. However, disease-modified ion channel function and pharmacological blockers (e.g., phenytoin and verapamil) may reduce NP permeability in HD brains (Nimigean and Allen, 2011; Zhou Y. et al., 2018).

Despite its benefits, passive targeting remains limited because the BBB’s restrictive structure prevents most hydrophilic and high-molecular-weight drugs from entering the brain, blocking approximately 98% of small molecules and 100% of large molecules. To overcome these barriers, strategies such as hyperosmotic disruption, microbubble-assisted ultrasound, and focused ultrasound (FUS) are under investigation to transiently open the BBB and improve nanoparticle delivery (Chu et al., 2022).

#### 10.1.2 Physical (invasive and non-invasive) techniques

Physical and physicochemical techniques aim to directly alter the BBB permeability to enhance drug delivery to the brain. The mannitol-induced osmotic disruption method is a classic invasive approach that temporarily dehydrates endothelial cells, widening tight junctions and increasing permeability (Fortin et al., 2007). However, excessive disruption may cause neuropathological damage, including myelin loss and irreversible central nervous system injury (Kozler et al., 2013).

In contrast, non-invasive methods, such as FUS, offer a safer, localized, and reversible way to transiently open the BBB. When combined with acoustically responsive microbubbles, FUS enhances drug diffusion into brain tissue. In HD preclinical models, FUS may improve delivery of neuroprotective agents to striatal regions while minimizing systemic exposure and immune activation. Preclinical studies have shown that FUS improved liposomal doxorubicin uptake and extended survival in glioma-bearing rats (Aryal et al., 2013) and increased boronophenylalanine-fructose (BPA-f) accumulation in gliosarcoma cells, supporting its use in boron neutron capture therapy (Alkins et al., 2013).

Magnetically guided nanoparticles (MNPs), particularly ultra-small superparamagnetic iron oxide (USPIO) nanoparticles integrated with microbubbles, can be driven across the BBB using transcranial ultrasound pulses and external magnetic fields (Huang et al., 2015). This synergistic approach enhances BBB permeability by up to 44% compared to osmotic disruption alone (Sun et al., 2014). Another innovative method, convection-enhanced delivery (CED), bypasses the BBB entirely by applying a controlled positive pressure gradient to infuse therapeutic agents directly into brain parenchyma (Foley et al., 2012). CED has demonstrated success with nano-liposomal irinotecan, achieving improved antitumor efficacy and prolonged survival in glioblastoma models (Chen et al., 2013). However, in HD, invasive strategies must carefully balance therapeutic benefit with risks of local inflammation and neuronal damage.

#### 10.1.3 Cell-penetrating peptides

Cell-penetrating peptides (CPPs) are short amino acid sequences (<30 residues) that enable cellular uptake of various drugs, from small molecules to large biomolecules (Cerrato et al., 2014). Classified by origin (natural, synthetic, and chimeric) and charge (cationic, amphiphilic, and hydrophobic), CPPs have been shown to cross the BBB effectively in both *in vitro* and *in vivo* studies (Zou et al., 2013). When conjugated with nanoparticles, CPPs improve drug delivery efficiency and stability. For instance, CPP-modified PLGA nanoparticles achieved nasal-to-brain insulin delivery with ∼6% brain uptake (Yan et al., 2013), while CPP-functionalized magnetic nanoparticles facilitated rapid cellular internalization via clathrin-mediated endocytosis (Chaudhary et al., 2013). In HD, CPPs may be particularly advantageous for delivering neurotrophic factors, small interfering RNA (siRNA), or small molecules directly to striatal neurons, overcoming both cellular and barrier-level transport limitations. However, proteolytic degradation by circulating enzymes limits their systemic stability and therapeutic longevity (Matsui et al., 2020).

#### 10.1.4 Active targeting

Active targeting utilizes receptor- and adsorption-mediated transcytosis (RMT and AMT). It is an energy-dependent processes that enable nanoparticle passage across the BBB (Grabrucker et al., 2016). Commonly targeted receptors include the transferrin receptor (TfR), insulin receptor (IR), and low-density lipoprotein receptor-related proteins (LRP1/2) (Han and Jiang, 2021). TfR-based targeting is widely applied, with transferrin-decorated liposomes and iron-mimetic peptides improving uptake into brain endothelial cells (Sharma et al., 2013). In HD, TfR-targeted nanocarriers may enhance the delivery of disease-modifying therapeutics to the striatum, potentially improving motor and cognitive outcomes.

The OX26 monoclonal antibody, which binds a distinct TfR epitope, allows delivery of therapeutics such as BDNF, achieving a 65%–70% reduction in stroke volume in preclinical studies (Zhang and Pardridge, 2006).

Insulin receptor-targeted nanoparticles, such as solid lipid nanoparticles (SLNs) conjugated with monoclonal antibodies, have enabled effective brain delivery of carmustine, resulting in strong anti-tumor results (Kuo and Shih-Huang, 2013). LRP1/2-mediated systems, including ligands such as lactoferrin, melanotransferrin, and Angiopep-2, also promote BBB penetration. For example, Angiopep-2-functionalized iron oxide nanoparticles have shown enhanced imaging and low cytotoxicity in glioma cells (Chen et al., 2014). Additional ligands such as RVG29 (from the rabies virus glycoprotein) facilitate clathrin- and caveolae-mediated endocytosis, thereby improving brain uptake of drugs such as camptothecin (Cook et al., 2015). Other nanocarriers, such as cyclodextrin (CD) and triphenylphosphonium (TPP)-conjugated nanoparticles, have achieved BBB passage via cholesterol extraction and lipophilic interactions, respectively (Vecsernyes et al., 2014; Han et al., 2020). Moreover, positively charged polymers (e.g., chitosan, polyethyleneimine, and poly-L-lysine) enhance adsorptive-mediated transcytosis, further broadening active transport potential (Zhi et al., 2021).

#### 10.1.5 Intranasal delivery

Intranasal drug administration provides a direct, non-invasive route to deliver drugs into the CNS, bypassing the BBB (Albash et al., 2025). Drugs can reach the brain through the olfactory and trigeminal nerve pathways, which connect the nasal mucosa to the CNS (Farag et al., 2025). In one study, methacrylic copolymer-functionalized poly (ε-caprolactone) nanocapsules loaded with olanzapine showed strong mucoadhesion and a 1.5-fold increase in brain concentration compared to conventional formulations (Fonseca et al., 2015). Although promising, intranasal delivery faces several physiological challenges, including enzymatic degradation, mucociliary clearance, and limited absorption surface area, as the olfactory region represents only approximately 5% of the nasal epithelium (Kim and Zhao, 2022). In HD, intranasal delivery may facilitate repeated, non-invasive administration of neuroprotective or gene-silencing therapeutics, bypassing both systemic exposure and BBB-related limitations. Enhancing formulation properties through mucoadhesive agents, absorption enhancers, and ligand decoration has shown potential to improve drug retention and CNS targeting (Taha et al., 2023). Table 8 summarizes the nano-technological approaches to overcome the BBB.

**TABLE 8:** Summary of nano-technological strategies to overcome the BBB.

Strategy	Mechanism of action	Key example/Fidning	Advantage	Limitation	References
Passive targeting	Energy-independent diffusion (simple, paracellular, or transcellular) exploiting pathophysiological differences (EPR effect)	AuNPs coated with PEG or TAT peptides cross BBB via diffusion; comparable to ion channels	Simple mechanism; selective accumulation in diseased tissues	Limited BBB permeability (blocks ∼98% small and 100% large molecules); may require adjunct BBB disruption (e.g., FUS and osmotic methods)	Nimigean and Allen (2011), Cheng et al. (2014), Wen and Meng (2014), Zhou et al. (2018b), Sharma et al., 2019; Wu (2021)
Physical/Physicochemical techniques	Direct modification of BBB permeability (invasive or non-invasive)	• Invasive: mannitol-induced osmotic opening widens tight junctions • Non-invasive: FUS + microbubbles enhance the delivery of liposomal doxorubicin and BPA-f. Magnetically guided USPIO + FUS improves BBB permeability by 44%. CED delivers drugs directly into brain tissue	Effective localized or controlled BBB opening; enhances drug penetration	Invasive methods risk CNS damage/toxin influx; non-invasive techniques need optimization	Fortin et al. (2007), Foley et al. (2012), Alkins et al. (2013), Aryal et al. (2013), Chen et al. (2013), Kozler et al. (2013), Sun et al. (2014), Huang et al. (2015)
Cell-penetrating peptides (CPPs)	Short peptides (<30 amino acids) enable translocation of drugs via endocytosis or direct penetration	CPP-modified PLGA NPs achieved ∼6% nasal-to-brain insulin delivery; CPP-functionalized magnetic NPs promote clathrin-mediated uptake	Versatile, applicable to various drugs; efficient BBB crossing	Susceptible to enzymatic degradation; limited systemic stability	Chaudhary et al. (2013), Yan et al. (2013), Zou et al. (2013), Cerrato et al. (2014), Matsui et al. (2020)
Active targeting	ATP-dependent receptor-/adsorption-mediated transcytosis (RMT/AMT)	• TfR (OX26–BDNF, 65%–70% stroke reduction) • IR (BCNU, anti-tumor efficacy) • LRP1/2 (Angiopep-2 NPs enhance imaging) • RVG29 improves camptothecin delivery • CD & TPP-NPs >> improve brain uptake via cholesterol/lipophilic interactions • Cationic polymers enhance brain uptake	Highly specific; enables targeted drug transport; adaptable to many ligands	Competitive binding with endogenous ligands; potential receptor saturation; complex design	Zhang and Pardridge (2006), Kuo and Shih-Huang (2013), Sharma et al. (2013), Chen et al. (2014), Vecsernyes et al. (2014), Cook et al. (2015), Han et al. (2020), Han and Jiang (2021)
Intranasal delivery	Direct transport via olfactory and trigeminal nerves, bypassing BBB	Methacrylic copolymer-functionalized PCL nanocapsules with olanzapine showed 1.5-fold higher brain concentration	Non-invasive; rapid brain delivery; avoids systemic circulation	Enzymatic degradation; mucociliary clearance; small olfactory area (∼5% of nasal mucosa)	Fonseca et al. (2015), Kim and Zhao (2022), Albash et al. (2025), Farag et al. (2025)

### 10.2 Advances in nanostructured sensors for HD diagnosis

Recent advances in nanotechnology have revolutionized the design and application of nanosensors for the early and precise diagnosis of neurodegenerative disorders, including HD. These cutting-edge diagnostic platforms integrate nanoscale materials with remarkable optical, electrical, and biochemical responsiveness to detect disease-related biomarkers with high sensitivity and selectivity (Shahrukh et al., 2025). Typically, nanosensors are composed of functional nanostructures such as plasmonic metals (gold and silver), fluorescent materials (quantum dots and carbon dots), or electroactive carbon-based nanomaterials (graphene and carbon nanotubes) (Zheng and Tan, 2020). A defining feature of these sensors is that their active sensing element operates at the nanometer scale, allowing the detection of even trace levels of biological or chemical signals. In most configurations, nanoparticles are conjugated with specific targeting ligands, such as antibodies, peptides, or aptamers, that selectively bind to the biomarker of interest. This combination provides biological specificity, while the nanoparticle core functions as a signal transducer or amplifier, converting the recognition event into a measurable optical or electrochemical response (Swierczewska et al., 2012).

**FIGURE 4:**
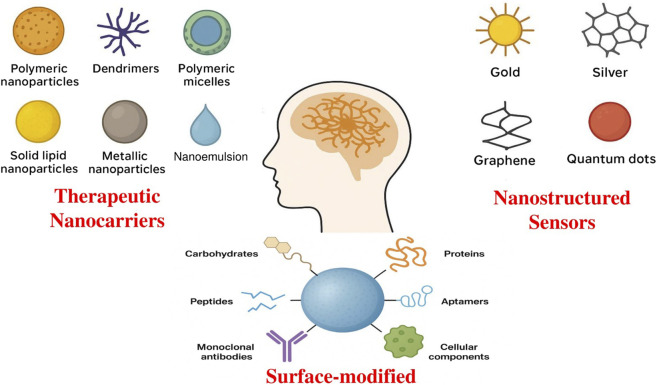
Schematic summary of nanotechnology-based approaches for HD, highlighting diagnostic nano-sensors for early biomarker detection, therapeutic nanocarriers for targeted drug delivery, and surface-modified techniques designed to enhance BBB penetration.

In the context of HD, which is characterized by the progressive loss of neurons in critical brain regions, nanosensors hold immense diagnostic promise. By identifying molecular alterations associated with HD at early stages, these nanosystems could enable timely intervention, improved disease monitoring, and the discovery of new therapeutic targets, thus contributing to a deeper understanding of the disease’s underlying pathophysiology. Figure 4 summarizes nano-technological advancements in the diagnosis and treatment of HD.

### 10.3 Nanocarriers in HD therapy

Nanoparticles have gained increasing attention due to their ability to carry large amounts of drugs, show minimal systemic toxicity, and maintain excellent physical and chemical stability while enhancing the therapeutic performance of loaded compounds (Taha et al., 2023; Ahmed et al., 2025a; Tawfik et al., 2025). Their ability to cross the BBB is strongly influenced by factors such as size, polarity, surface chemistry, and particle type (Tawfik et al., 2023). By attaching biomolecules or ligands, nanoparticles can safely and effectively deliver pharmacologically active agents, including drugs or genes, across physiological barriers (Albash et al., 2025). Typically ranging between 1 and 100 nm, these nanocarriers can be further modified with polymers or targeting ligands to improve binding affinity and delivery efficiency. For neurodegenerative disorders, including HD, nanocarriers sized 20–100 nm, are considered ideal. Moreover, gene-based nanotherapies using siRNA have shown promise in silencing the HTT gene, thereby reducing the expression of the defective protein associated with HD (Jagaran and Singh, 2021).

#### 10.3.1 Solid lipid nanoparticles

Solid lipid nanoparticles (SLNs), typically ranging from 10 to 1,000 nm, are biocompatible lipid-based nanocarriers made of fatty acids, waxes, or triglycerides (Satapathy et al., 2021). They exhibit high drug-loading capacity, large surface area, and excellent stability, which enable efficient delivery of both hydrophilic and lipophilic drugs, peptides, and genes (Tapeinos et al., 2017). In HD therapy, curcumin-loaded SLNs improved drug bioavailability and provided strong antioxidant and anti-inflammatory neuroprotection, thereby reducing neuronal damage (Cao and Zhang, 2022). Similarly, rosmarinic acid-loaded SLNs delivered intranasally improved motor coordination and reduced oxidative stress in HD rat models (Bhatt et al., 2015). Thymoquinone-loaded SLNs restored mitochondrial ATPase function and suppressed neuro-inflammation, producing superior neuroprotective effects at lower doses (Ramachandran and Thangarajan, 2018).

#### 10.3.2 Polymeric nanoparticles

Polymeric nanoparticles (PNPs) (1–1,000 nm) are formulated from synthetic or natural polymers, allowing drugs to be loaded through surface adsorption, encapsulation, or chemical attachment (Patel et al., 2012). Their controlled-release properties, high biocompatibility, and structural flexibility make them ideal for targeted therapy (El-Say and El-Sawy, 2017). Experimental studies confirm the potential of PNPs for HD. PNPs restored synaptic and cognitive function in R6/2 HD mice by efficiently delivering neuroprotective agents (Siafaka et al., 2022), while chitosan-based nanoparticles carrying anti-HTT siRNA suppressed mutant HTT gene expression in YAC128 mice (Sava et al., 2020).

#### 10.3.3 Dendrimers

Dendrimers are highly branched, tree-like nanostructures with precise architecture and multiple surface functionalities, enabling them to encapsulate or conjugate a wide range of therapeutic agents (Florendo et al., 2018). Their high surface area supports efficient transport of hydrophobic drugs across the BBB, improving solubility and bioavailability. Among dendrimers, polyamidoamine exhibits strong biocompatibility and BBB permeability, whereas polylysine dendrimers promote efficient cellular uptake, making them suitable for gene therapy applications in neurodegenerative disorders (Mignani et al., 2017).

#### 10.3.4 Polymeric micelles

Formed by the self-assembly of amphiphilic copolymers, polymeric micelles create a hydrophobic core that encapsulates poorly soluble drugs and a hydrophilic shell that enhances stability and circulation time (Ahmed et al., 2023). Depending on morphology, they can be spherical, vesicular, or irregularly shaped (Ahmed et al., 2021; Teama et al., 2025). In HD-related research, polymeric micelles have been shown to prevent polyglutamine aggregation in HD150Q cell models, suggesting their capacity to inhibit mutant huntingtin protein accumulation and reduce neuronal toxicity (Debnath et al., 2019).

#### 10.3.5 Liposomes

Liposomes are spherical vesicles with phospholipid bilayers enclosing an aqueous core, allowing simultaneous encapsulation of hydrophilic and hydrophobic drugs (Liu et al., 2022). They have demonstrated success in gene therapy, vaccine delivery, and anticancer treatments. Studies have shown that liposomal siRNA formulations efficiently suppress target gene expression in various cell lines (Mantha et al., 2012). Moreover, apo-lipoprotein E (Apo-E)-peptide-based curcumin liposomes effectively penetrated the BBB and protected curcumin from degradation, enhancing its neuroprotective efficacy in HD (Vishwas et al., 2021).

#### 10.3.6 Metallic nanoparticles

Metallic nanoparticles, including gold, silver, selenium, and magnetic nanosystems, are widely used for drug delivery and neuroimaging due to their large surface area and tunable reactivity (Chandrakala et al., 2022). In HD studies, selenium nanoparticles (nano-Se) reduced oxidative stress, inhibited mHTT aggregation, and downregulated histone deacetylase gene expression, providing significant neuroprotection (Cong et al., 2019). Similarly, trehalose-functionalized gold nanoparticles (20–30 nm) prevented toxic protein aggregation, increased neuronal survival, and showed strong therapeutic potential for HD (Mandal et al., 2017).

#### 10.3.7 Hydrogels

Hydrogels are three-dimensional, water-rich polymer networks capable of incorporating a wide range of polymers such as PVA, PEO, PNVP, and polysaccharides. They can be designed for localized or systemic delivery of drugs targeting neurodegenerative disorders (Mukherjee et al., 2020). An embelin-loaded *in situ* hydrogel (20% Pluronic F-127 + 0.3% Carbopol 934) demonstrated controlled drug release and enhanced brain targeting after intranasal delivery, significantly improving oxidative and behavioral markers in HD rats (Shahrukh et al., 2025).

#### 10.3.8 Nanoemulsions

Nanoemulsions (NEs) are lipid-based colloidal dispersions that enhance drug solubility and bioavailability while easily crossing the BBB. They can be prepared by high-energy (sonication and homogenization) or low-energy techniques (Nirale et al., 2020). In HD models, tetrabenazine-loaded NEs improved drug permeability by 1.68-fold, bypassed first-pass metabolism via intranasal administration, and markedly increased brain drug concentration, leading to better control of hyperkinetic movements compared to conventional formulations (Arora et al., 2020). A comparison of nanocarrier-based formulations in HD is presented in Table 9.

**TABLE 9:** Comparative summary of nanocarrier-based formulations in HD.

Formulation type	Key feature	Advantage	Limitation	Example in HD	Translational stage	References
Solid lipid nanoparticles (SLNs)	Lipid-based nanocarriers (10–1,000 nm)	High drug loading, stability, biocompatibility	Limited drug expulsion, scale-up issues	Curcumin SLNs improved neuroprotection	Preclinical	Sandhir et al. (2014), Gharaibeh et al. (2020)
Polymeric nanoparticles (PNPs)	Polymer-based controlled-release systems	Tunable release, high biocompatibility	Potential toxicity depending on polymer	siRNA-loaded chitosan nanoparticles reduced mHTT	Preclinical	Mendonça et al. (2021)
Liposomes	Phospholipid vesicles	Carry hydrophilic and lipophilic drugs	Stability and leakage issues	ApoE-curcumin liposomes enhanced BBB penetration	Preclinical/early clinical	Seko et al. (2020)
Polymeric micelles	Amphiphilic self-assembled systems	Improve solubility of hydrophobic drugs	Limited drug-loading capacity	Reduced polyQ aggregation	Preclinical	Leitman et al. (2013)
Metallic nanoparticles (MNPs)	Gold, silver, selenium NPs	Imaging + therapeutic potential	Long-term toxicity concerns	Nano-Se reduced oxidative stress	Preclinical	Cong et al. (2019)
Nanoemulsions (NEs)	Lipid colloidal dispersions	Improve solubility, BBB penetration	Stability issues	Tetrabenazine NE improved brain delivery	Preclinical	Arora et al. (2020)
Hydrogels	3D polymer networks	Controlled release, local delivery	Limited penetration alone	Embelin hydrogel improved outcomes	Preclinical	Kapahi et al. (2020)

### 10.4 Surface modification of nanoparticles for enhanced permeation through the BBB

The functional modification of nanoparticle surfaces with biological ligands such as proteins, carbohydrates, peptides, aptamers, monoclonal antibodies, and cellular membranes has emerged as a powerful strategy to overcome the BBB and achieve selective brain targeting. Each ligand type interacts with specific transporters or receptors, promoting receptor-mediated endocytosis and improving drug delivery efficiency for neurodegenerative disease treatment (Pramoda et al., 2025).

#### 10.4.1 Protein-functionalized nanoparticles

Proteins serve as natural targeting ligands owing to their receptor specificity and biocompatibility. Transferrin (Tf) and lactoferrin (Lf), for example, exploit the upregulated iron transport system in neurodegenerative diseases to mediate BBB translocation. Tf-coated nanolipid carriers have enhanced brain uptake of therapeutic agents such as rivastigmine and resveratrol by 1.7-fold compared to unmodified systems (Jain et al., 2023). Similarly, Lf-conjugated nanosystems effectively delivered linoleic acid across the BBB, exerting antioxidant effects in Alzheimer’s models (Agwa et al., 2020). Apo-E-coated nanoparticles also utilize lipoprotein receptor pathways to cross the BBB and reduce amyloid aggregation (Song et al., 2014). Exosome-based protein modifications, such as LFA-1-tagged vesicles, further enhance curcumin transport by binding endothelial ICAM-1, mitigating tau phosphorylation, neuronal apoptosis, and potential mutant huntingtin toxicity (Wang et al., 2019).

#### 10.4.2 Carbohydrate-modified nanocarriers

Carbohydrates, such as glucose, mannose, and lactose, act as efficient targeting moieties by mimicking endogenous sugar transport systems. Glucose-modified liposomes carrying quercetin exhibited a 3.3-fold higher BBB permeability and stronger antioxidant protection compared to unmodified formulations (Chen et al., 2024). Mannose-functionalized nanoparticles target mannose receptors on microglia and endothelial cells, promoting anti-inflammatory and neuroprotective effects through M2 polarization and phagocytosis of toxic aggregates (Lei et al., 2024). Likewise, lactose-modified nanocarriers enhance drug accumulation in brain tissue, offering a promising route for controlled CNS delivery (Yokoyama et al., 2020).

#### 10.4.3 Peptide-grafted nanoparticles

Peptide ligands provide high specificity and tunable affinity for BBB transporters. The g7 peptide, a glycopeptide derivative, facilitates nanoparticle uptake via macro-pinocytosis and has been used to restore cholesterol metabolism and cognitive performance in HD models (Birolini et al., 2021). Similarly, RVG, derived from the rabies virus glycoprotein, guides siRNA-loaded nanoparticles across brain endothelial barriers for gene silencing of the mHTT gene (Mendonca et al., 2021). Other peptides, including T7 and FGL, bind Tf or fibroblast growth factor receptors to enhance siRNA and neuroprotective peptide delivery, thereby suppressing amyloid aggregation, tau phosphorylation, and potential neurodegeneration associated with polyglutamine expansion in HD (Qian et al., 2022). Leptin-derived and tetanus toxin fragment (TTC) peptides are also explored for receptor-mediated BBB transport and neuro-regeneration (Harvey, 2024).

#### 10.4.4 Aptamer-conjugated systems

Aptamers are short, single-stranded DNA or RNA oligonucleotides that act as artificial antibodies with excellent binding precision. Their chemical flexibility enables conjugation with nanoparticles for targeted brain drug delivery. Aptamer-linked gold nanoparticles have been designed to bind amyloid-β (Aβ_1_–_40_), thereby blocking fibril formation and reducing cytotoxicity (Qin et al., 2022). Similarly, aptamer-functionalized PLGA nanoparticles improved the site-specific delivery of curcumin and enhanced its therapeutic response (Mathew et al., 2012). Novel aptamers such as Apt19S, which recognize neural stem cells, have been integrated into nanocarriers for dopaminergic neuron regeneration and reduction of neuroinflammation, representing an innovative approach for Parkinson’s therapy (Sun et al., 2023).

#### 10.4.5 Monoclonal antibody-modified nanocarriers

Monoclonal antibodies (mAbs) enable highly specific, receptor-guided drug transport across the BBB. OX26, a TfR–binding mAb, has been widely used to ferry dopamine or peptides to the brain, showing an eightfold increase in BBB penetration compared to free drugs (Kang et al., 2016). Dual-targeted nanocarriers combining OX26 and HD-specific antibodies could potentially enhance mutant huntingtin clearance while improving neuroprotection in HD models (Loureiro et al., 2016). Such antibody-guided nanocarriers combine molecular precision with enhanced stability, offering an ideal platform for neurodegenerative therapy.

#### 10.4.6 Cell- and membrane-derived nanocarriers

Leveraging biological membranes from red blood cells, macrophages, or neural stem cells enables stealth-like behavior and receptor-mediated targeting. Macrophage-membrane-coated nanoparticles functionalized with RVG29 and triphenylphosphonium (TPP) achieved superior BBB transport, mitochondrial localization, and neuroprotection (Han et al., 2022). Similarly, RVG-overexpressing neural stem cell membranes facilitated bexarotene delivery and significantly reduced β-amyloid levels (Huang D. et al., 2023). Red blood cell membrane-coated up-conversion nanoparticles loaded with nitric oxide donors enhanced striatal drug bioavailability and exerted anti-inflammatory effects, suggesting promise for HD therapy (Huang Z. et al., 2023). The nano-technological advancements in the diagnosis and treatment of HD are summarized in Table 10.

**TABLE 10:** Summary of nano-technological advancements in the diagnosis and treatment of HD.

Nanosystems	Category	Nano-platform/Example	Composition/Core material	Mechanism or function	Key outcomes in HD or neurodegenerative models	References
Nanostructured sensors	For diagnosis	Plasmonic metals, quantum dots, carbon nanotubes, graphene	Gold, silver, fluorescent or carbon-based nanomaterials	Detect HD biomarkers via optical, electrochemical, or fluorescence signals	Early and sensitive detection of HD-related molecular changes enabling timely diagnosis	Swierczewska et al. (2012), Zheng and Tan (2020), Shahrukh et al. (2025)
Therapeutic nanocarriers	Solid lipid nanoparticles (SLNs)	Fatty acids, waxes, triglycerides	Biocompatible lipids (10–1,000 nm)	Encapsulate hydrophobic/lipophilic drugs for sustained release	Curcumin and rosmarinic acid SLNs improved antioxidant activity and reduced neuronal loss	Bhatt et al. (2015), Tapeinos et al. (2017), Ramachandran and Thangarajan (2018), Satapathy et al. (2021), Cao and Zhang (2022)
	Polymeric nanoparticles (PNPs)	PLGA, chitosan, PEG polymers	Synthetic/natural polymers	Controlled drug release and siRNA delivery	Suppressed mHTT expression; restored synaptic and cognitive functions	Patel et al. (2012), El-Say and El-Sawy (2017), Sava et al. (2020), Siafaka et al. (2022)
	Dendrimers	Polyamidoamine, polylysine	Branched polymeric architecture	Surface-functionalized carriers enhancing BBB transport	Improved gene and drug delivery; reduced aggregation toxicity	Mignani et al. (2017), Florendo et al. (2018)
	Polymeric micelles	Amphiphilic block copolymers	Hydrophobic core, hydrophilic shell	Solubilize and deliver poorly soluble drugs	Prevented polyglutamine aggregation in HD cell models	Debnath et al. (2019), Ahmed et al. (2021), Ahmed et al. (2023), Teama et al. (2025)
	Liposomes	Phospholipid bilayer vesicles	Hydrophilic core + hydrophobic membrane	Dual drug encapsulation, prolonged circulation	Apo-E–curcumin liposomes enhanced BBB transport and neuroprotection	Mantha et al. (2012), Vishwas et al. (2021), Liu et al. (2022)
	Metallic nanoparticles (MNPs)	Gold, selenium, silver, magnetic nanoparticles	Metal or metal oxide cores	Antioxidant and anti-aggregation actions	Nano-Se and trehalose AuNPs reduced oxidative stress and mHTT aggregation	Mandal et al. (2017), Cong et al. (2019), Chandrakala et al. (2022)
	Hydrogels	PVA, PEO, PNVP, Carbopol	3D polymeric networks	Controlled intranasal drug release	Embelin hydrogel enhanced oxidative balance and behavioral recovery in HD rats	Mukherjee et al. (2020), Shahrukh et al. (2025)
	Nanoemulsions (NEs)	Oil/water colloids (lipid-based)	Surfactant-stabilized nanodroplets	Enhance solubility, BBB penetration	Tetrabenazine NEs improved brain uptake and motor control	Arora et al. (2020), Nirale et al. (2020)
Surface modification of nanoparticles	Protein-functionalized nanoparticles	Transferrin, lactoferrin, Apo-E, exosome proteins	Protein–nanoparticle conjugates	Receptor-mediated endocytosis via Tf/Lf/Apo-E pathways	Enhanced brain uptake (1.7–2 x), reduced oxidative and amyloid pathology	Song et al. (2014), Wang et al. (2019), Agwa et al. (2020), Jain et al. (2023)
	Carbohydrate-modified nanoparticles	Glucose, mannose, lactose	Sugar–nanoparticle conjugates	Utilize GLUT and mannose receptor transport	Improved BBB permeability (up to 3.3 x); promoted anti-inflammatory activity	Yokoyama et al. (2020), Chen et al. (2024), Lei et al. (2024)
	Peptide-grafted nanoparticles	g7, RVG, T7, FGL, TTC	Peptide–polymer or peptide–lipid conjugates	Target specific BBB transporters	Restored cholesterol metabolism and reduced mHTT aggregation	Birolini et al. (2021), Mendonca et al. (2021), Qian et al., 2022; Harvey (2024)
	Aptamer-conjugated systems	DNA/RNA aptamers	Aptamer–gold or polymer hybrid	High-affinity binding to disease-specific proteins	Blocked Aβ fibril formation, improved curcumin delivery, enhanced neuroprotection	Mathew et al. (2012), Qin et al. (2022), Sun et al. (2023)
	Monoclonal antibody–modified nanoparticles	OX26 and DE2B4	Antibody–lipid or antibody–polymer conjugates	Dual-targeting of BBB receptors and pathogenic proteins	8x increase in brain uptake; strong amyloid inhibition	Kang et al. (2016), Loureiro et al. (2016)
	Cell-/Membrane-derived nanoparticles	RBC, macrophage, neural stem cell membranes	Biomimetic nanocarriers	Immune evasion + receptor-mediated targeting	RVG–TPP–macrophage nanoparticles improved BBB transport, mitochondrial targeting, and neuroprotection	Han et al. (2022)

## 11 Emerging therapeutic frontiers in HD: genetic and regenerative innovations

### 11.1 Genetic innovations

Modern advances in gene-editing technologies offer transformative potential for tackling genetic neurodegenerative disorders such as HD. Precision editing tools such as CRISPR/Cas9, base editing, and the more refined PRIME editing system are being explored to correct or silence the mutated gene directly, aiming to suppress mHTT synthesis at its source (Chavhan et al., 2025). These approaches may ultimately enable one-time, disease-modifying therapies that halt or even reverse neurodegeneration.

#### 11.1.1 CRISPR/Cas9-mediated editing

The CRISPR/Cas9 system, derived from bacterial immune defense mechanisms, remains the cornerstone of genome editing. Guided by RNA sequences (gRNA), the Cas9 enzyme introduces targeted double-stranded DNA breaks (DSBs) within the genome (Aswendt et al., 2015; Zhou X. et al., 2018). Early HD studies used RNA interference (RNAi) or antisense oligonucleotides (ASOs) to degrade mHTT mRNA, thereby reducing toxic protein accumulation (Kingwell, 2021). However, CRISPR/Cas9 surpasses these by permanently modifying the DNA sequence, thereby silencing mutant gene expression (Li et al., 2019). Animal model research confirms its therapeutic promise: unique single-nucleotide polymorphisms (SNPs) have successfully deleted mutant alleles of HTT, while non-allele-specific gRNAs targeting the CAG repeat region have reduced mHTT toxicity and improved motor and cognitive functions in HD mice (Wang et al., 2016; Monteys et al., 2017). Despite its precision, CRISPR/Cas9 faces challenges such as unintended DSB-induced errors and off-target mutations, which may alter essential genes. Strategies like limiting Cas9 exposure and employing engineered high-fidelity variants (e.g., Sniper-Cas9) are improving safety and accuracy (Guan et al., 2022).

#### 11.1.2 PRIME editing: precision without breaks

PRIME editing represents a next-generation evolution of CRISPR/Cas9, designed for greater control and minimal DNA damage. Another technology combines a Cas9 nickase fused with a reverse transcriptase enzyme and a specialized guide RNA (pegRNA) to “search and replace” specific DNA bases without generating DSBs (Matsoukas, 2020). This system reduces off-target effects, minimizes insertions/deletions, and enables accurate base substitutions, insertions, or deletions at nearly any genomic site (Jiang and Yao, 2022). Though not yet applied directly to HD models, PRIME editing holds strong potential to selectively modulate HTT gene expression by precisely targeting CAG expansions or regulatory regions (Kantor et al., 2020). Its high fidelity, reduced mutagenic risk, and ability to perform non-destructive gene correction position PRIME editing as a future cornerstone for gene therapy in HD.

### 11.2 Stem cell–based regenerative strategies

Stem cell therapy has emerged as one of the most promising regenerative approaches for neurodegenerative diseases, particularly HD. By replacing damaged neurons or restoring neurotrophic support, stem cells offer a restorative path that traditional treatments cannot achieve (De Gioia et al., 2020). Unlike conventional pharmacological approaches that provide symptomatic relief, stem cell-based interventions can potentially regenerate neuronal networks and restore lost functions.

#### 11.2.1 Stem cell sources and mechanisms

Various cell types as neural stem cells (NSCs), mesenchymal stem cells (MSCs), induced pluripotent stem cells (iPSCs), and embryonic stem cells (ESCs), are under investigation for HD therapy, each providing distinct regenerative benefits (Saha et al., 2022). MSCs, which can be isolated from bone marrow, adipose tissue, and umbilical cord blood, are particularly valued for their ease of harvesting, self-renewal capacity, and immunomodulatory effects (Crane et al., 2014). They secrete neurotrophic factors and cytokines that promote neurogenesis, angiogenesis, and synaptic repair while reducing neuroinflammation and apoptosis (Colpo et al., 2017). iPSCs, generated by reprogramming adult somatic cells into a pluripotent state, enable the creation of patient-specific neuronal cells for autologous transplantation, reducing immune rejection (Ye et al., 2013). Similarly, NSCs can be directly induced from fibroblasts using transcription factors such as SOX2 and PAX6, offering another source for generating functional neurons capable of restoring neural activity (Zhang and Cui, 2014).

#### 11.2.2 Current challenges and nanotechnology integration

Despite decades of progress, stem cell therapy for HD still faces key hurdles including ethical limitations (especially for ESC use), scalability, and precise control of cell fate *in vivo*. Tracking transplanted cells, ensuring correct neuronal differentiation, and achieving sustained integration into host circuits remain critical challenges (Adams et al., 2016). Nanotechnology is emerging as a synergistic tool to overcome these barriers. Nanomaterials, owing to their high surface area, biocompatibility, and unique optical and electrical properties, can enhance stem cell tracking, differentiation, and survival (Zhang et al., 2018). They can also serve as bioactive scaffolds, drug carriers, or imaging agents, thereby supporting stem cell proliferation and controlled neural regeneration. Functionalized nanoparticles, when combined with stem cell platforms, may ultimately improve targeted delivery and therapeutic precision within the brain (Zhou X. et al., 2018).

## 12 Regulatory and manufacturing challenges of orphan drugs

The orphan drug development landscape for HD represents a critical frontier in rare disease therapeutics, but it faces distinct regulatory and manufacturing hurdles stemming from the genetic and clinical complexity and rarity of the disorder. Emerging evidence highlights the role of epigenetic modulation that influences inflammatory signaling pathways, cellular differentiation, and disease progression (Abdel Mageed et al., 2024; Sayed et al., 2025). Despite advances in molecular understanding of HD and emerging therapeutic strategies—such as antisense oligonucleotides (ASOs), siRNAs, gene editing, and more traditional small molecules, bringing treatments from bench to bedside remains challenging (Burgunder, 2015).

One overarching regulatory challenge for orphan drugs in HD arises from the disease’s rarity and heterogeneity. Regulatory agencies such as the U.S. Food and Drug Administration (FDA) and the European Medicines Agency (EMA) provide orphan drug designations to incentivize development based on criteria including low prevalence and unmet medical need. However, limited patient populations complicate clinical trial design—making randomized controlled trials with traditional endpoints difficult. HD’s long prodromal phase and variable progression necessitate sensitive, disease-specific biomarkers and clinical endpoints to demonstrate efficacy. For example, measuring reduction in mHTT protein or improvements in motor score may not capture subtle disease modifications within a timeframe feasible for clinical trials (Boat and Field, 2011; Yoo, 2024).​

Moreover, regulatory reviewers may have inconsistent interpretations of acceptable surrogate endpoints and trial designs tailored to small cohorts. Resources for engaging in early and ongoing dialog with sponsors to align on study protocols can be insufficiently allocated, particularly within divisions unfamiliar with rare diseases, leading to variability and delays in approval processes. The lack of standardized outcome measures further complicates the assessment of benefit–risk balance for orphan HD drugs. The push for reasoned flexibility in regulatory standards acknowledges that applying conventional criteria may not be feasible and that alternative evidence frameworks should be embraced, although consensus on this remains evolving (Boat and Field, 2011).

The innovative therapeutic modalities for HD, including ASOs and gene therapies targeting HTT mRNA, introduce specific regulatory concerns, such as assessing the long-term safety of gene silencing and off-target effects. Delivery mechanisms (e.g., intracerebroventricular administration) pose additional regulatory scrutiny due to invasiveness and drug distribution challenges. Balancing expedited access for a devastating and irreversible disease with rigorous evaluation of safety and efficacy is a persistent tension in orphan drug regulation for HD (Burgunder, 2015).

From a manufacturing perspective, orphan HD drugs tend to be highly complex biologics or nucleic acid-based therapies that demand sophisticated production capabilities. ASOs and siRNAs require consistent synthesis of specific sequences with stringent purity, stability, and chemical modifications to ensure durability and biological activity. Manufacturing scale-up is challenging due to the limited market size, requiring specialized facilities often tailored for small-batch production under Good Manufacturing Practice (GMP) compliance. This raises costs and limits availability (Yoo, 2024).​

Gene therapies for HD leverage viral vectors for delivery, which necessitate complex upstream and downstream manufacturing processes, including vector design, vector genome packaging, and purification. Controlling batch-to-batch variability, ensuring vector potency, and minimizing adventitious agents are critical quality considerations. Furthermore, storage and distribution logistics for biologics and gene therapies often require cold chain systems, increasing operational complexity. The high cost of production is partly passed to patients, contributing to the substantial economic burden associated with orphan drugs (Yoo, 2024).​

In addition, the administration routes for HD therapies, often intrathecal or intracerebral, increase the need for coordination between manufacturing and clinical delivery. The short shelf-life of some biologics limits inventory flexibility, requiring precise manufacturing and treatment scheduling.

## 13 Conclusion

While neuronal degeneration remains the hallmark of HD, emerging evidence highlights significant non-neuronal contributions, including immune dysregulation, gut microbiota alterations, and peripheral organ involvement. Innovative therapeutic strategies, such as nanotechnology-based delivery systems, non-invasive approaches (e.g., intranasal or transdermal), repurposed drugs, and improved experimental models, offer promising avenues to enhance treatment efficacy.

Despite these advances, important gaps remain. Preclinical models often fail to fully recapitulate human HD; the underlying disease mechanisms are not completely understood, and translating therapies into the clinic is constrained by biological, manufacturing, and regulatory challenges. Sex-specific differences, environmental factors, and lifestyle influence further complicate treatment optimization.

Taken together, addressing these challenges requires integrative, precision-medicine approaches that combine advanced drug delivery, gene and biologic therapies, patient-specific genetic and microbiome profiling, and lifestyle interventions. Incorporating sex-informed strategies, non-invasive monitoring, and targeted nanomedicine has the potential to improve therapeutic outcomes and slow disease progression. Altogether, these approaches provide a roadmap for future HD research and the development of individualized, translationally relevant treatments.

## Glossary

**3-NP**: 3-Nitropropionic acid
**AMPK**: AMP-activated protein kinase
**Apo-E**: Apolipoprotein E
**ASOs**: Antisense oligonucleotide(s)
**AuNPs**: Gold nanoparticles
**BBB**: Blood–brain barrier
**BCSFB**: Blood–cerebrospinal fluid barrier
**BDNF**: Brain-derived neurotrophic factor
**BPA-F**: Boronophenylalanine-fructose
**Cas9**: CRISPR-associated protein 9
**CED**: Convection-enhanced delivery
**CNS**: Central nervous system
**CP**: Choroid plexus
**CPPs**: Cell-penetrating peptide(s)
**CSF**: Cerebrospinal fluid
**DSBs**: Double-stranded DNA breaks
**EMA**: European Medicines Agency
**EPR**: Enhanced permeability and retention
**ESCs**: Embryonic stem cells
**FDA**: Food and Drug Administration
**FUS**: Focused ultrasound
**GMP**: Good Manufacturing Practice
**HD**: Huntington’s disease
**iPSCs**: Induced pluripotent stem cell(s)
**IKKγ**: IκB kinase γ-subunit
**IL**: Interleukin
**IR**: Insulin receptor
**Lf**: Lactoferrin
**LRP1/2**: Low-density lipoprotein receptor-related proteins 1/2
**MNPs**: Magnetically guided/metallic nanoparticle(s)
**mHTT**: Mutant huntingtin
**mMS**: Modified motor score
**MSH3**: MutS homolog 3
**MSCs**: Mesenchymal stem cells
**NEs**: Nanoemulsion(s)
**NK-κB**: Nuclear factor-κB
**NSCs**: Neural stem cells
**PEG**: Polyethylene glycol
**PNPs**: Polymeric nanoparticle(s)
**PolyQ**: Polyglutamine
**QA**: Quinolinic acid
RNAi: RNA interference
**RVG29**: Rabies virus glycoprotein 29
**siRNA**: Small interfering RNA
**SLNs**: Solid lipid nanoparticle(s)
**SNPs**: Single-nucleotide polymorphisms
**TAT**: Trans-activator of transcription
**Tf**: Transferrin
**TfR**: Transferrin receptor
**TFC**: Total functional capacity
**TMC score**: Total maximal chorea score
**TNF-α**: Tumor necrosis factor-α
**TPP**: Triphenylphosphonium
**TTC**: Tetanus toxin fragment
**UHDRS**: Unified HD rating scale
**USPIO**: Ultra-small superparamagnetic iron oxide
**VMAT2**: Vesicular monoamine transporter type 2
