# Supplementary figures and images for: An integrative neuropharmacological review of Huntington’s disease challenges and the role of novel formulations in addressing pharmacological‒pharmaceutical limitations
Source: Front Pharmacol. 2026 May 7;17:1794983. doi: 10.3389/fphar.2026.1794983 (PMC13190188; doi:10.3389/fphar.2026.1794983)

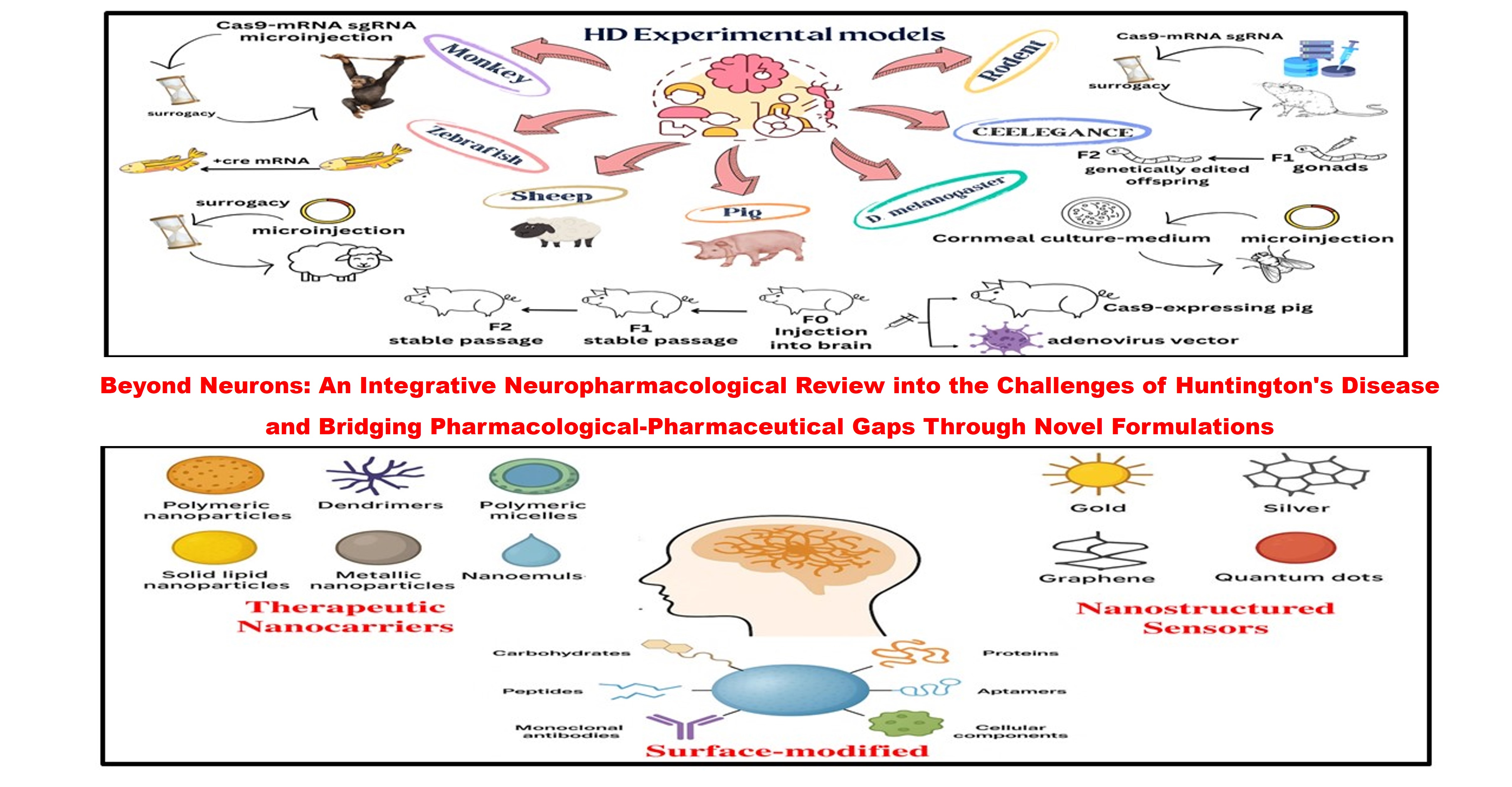

Supplement: Supplementary file 1 [file Image1.jpeg]
